# Defining Mononuclear Phagocyte Subset Homology Across Several Distant Warm-Blooded Vertebrates Through Comparative Transcriptomics

**DOI:** 10.3389/fimmu.2015.00299

**Published:** 2015-06-19

**Authors:** Thien-Phong Vu Manh, Jamila Elhmouzi-Younes, Céline Urien, Suzana Ruscanu, Luc Jouneau, Mickaël Bourge, Marco Moroldo, Gilles Foucras, Henri Salmon, Hélène Marty, Pascale Quéré, Nicolas Bertho, Pierre Boudinot, Marc Dalod, Isabelle Schwartz-Cornil

**Affiliations:** ^1^UM2, Centre d’Immunologie de Marseille-Luminy, Aix Marseille Université, Marseille, France; ^2^U1104, INSERM, Marseille, France; ^3^UMR7280, CNRS, Marseille, France; ^4^UR892, Virologie et Immunologie Moléculaires, INRA, Domaine de Vilvert, Jouy-en-Josas, France; ^5^IFR87 La Plante et son Environnement, IMAGIF CNRS, Gif-sur-Yvette, France; ^6^CRB GADIE, Génétique Animale et Biologie Intégrative, INRA, Domaine de Vilvert, Jouy-en-Josas, France; ^7^UMR1225, Université de Toulouse, INPT, ENVT, Toulouse, France; ^8^UMR1225, Interactions Hôtes-Agents Pathogènes, INRA, Toulouse, France; ^9^UMR1282, Infectiologie et Santé Publique, INRA, Nouzilly, France; ^10^UMR1282, Université François Rabelais de Tours, Tours, France

**Keywords:** comparative biology, immunology, dendritic cells, monocytes, macrophages, genomic and bio-informatic methods

## Abstract

Mononuclear phagocytes are organized in a complex system of ontogenetically and functionally distinct subsets, that has been best described in mouse and to some extent in human. Identification of homologous mononuclear phagocyte subsets in other vertebrate species of biomedical, economic, and environmental interest is needed to improve our knowledge in physiologic and physio-pathologic processes, and to design intervention strategies against a variety of diseases, including zoonotic infections. We developed a streamlined approach combining refined cell sorting and integrated comparative transcriptomics analyses which revealed conservation of the mononuclear phagocyte organization across human, mouse, sheep, pigs and, in some respect, chicken. This strategy should help democratizing the use of omics analyses for the identification and study of cell types across tissues and species. Moreover, we identified conserved gene signatures that enable robust identification and universal definition of these cell types. We identified new evolutionarily conserved gene candidates and gene interaction networks for the molecular regulation of the development or functions of these cell types, as well as conserved surface candidates for refined subset phenotyping throughout species. A phylogenetic analysis revealed that orthologous genes of the conserved signatures exist in teleost fishes and apparently not in Lamprey.

## Introduction

Reaching the global health objective requires to improve disease prevention and treatments in humans and in a wide variety of animal species. To achieve that goal, knowledge of the immune system, and particularly of the mononuclear phagocyte system that orchestrates the immune response, needs to be translated across species in order to develop better vaccines and immune response-targeting therapies in relevant species.

The mononuclear phagocytes encompass three main functional cell types: monocytes (Mo), macrophages (MP), and DC. The main functions of Mo are to patrol the body to detect infections and to produce microbicidal compounds including TNF, superoxide, or nitric oxide intermediates, or to differentiate into MP. The main function of MP is to preserve tissue homeostasis through trophic and scavenger functions. DCs are professional antigen-presenting cells that are key instructors of immunity, controlling tolerance to self and immune defense against pathogens. However, beyond these generic definitions, each of these mononuclear phagocyte category encompasses a complex array of different subtypes with distinct ontogeny and functions, as described extensively in mice and to some extent in humans. Mo include at least two main subsets, classical Mo (cMo) and non-classical Mo (ncMo) (1), that express different innate immune recognition receptors and mediate distinct functions, with ncMo showing the original property of patrolling blood vessels (2). Adult MP are derived either from embryonic precursors and self-renew in tissues, or in some cases are replenished from circulating Mo (2–6). The MP subtypes populating different tissues show distinct molecular and functional characteristics which are in a large part determined by their anatomical microenvironment (7, 8). Two cell types with morphologic and functional features of DC derive from the Mo/MP lineage, namely monocyte-derived DC (MoDC) and Langerhans cells (9). MoDC are generated (i) upon inflammatory stimuli *in vivo* (10), (ii) at steady-state in the skin (3), and (iii) upon culture of purified Mo or of total bone marrow cells with GM-CSF ± IL-4 *in vitro* (11, 12). Langerhans cells derive from embryonic monocytic precursors upon IL-34 signaling and populate the outer layer of epithelia (13). Finally, three types of *bona fide* DC exist, the plasmacytoid DC (pDC) and the conventional DC (cDC) cDC1 and cDC2 types which derive from a bone marrow common DC precursor and are present both in lymphoid organs and as interstitial DC in the parenchyma of non-lymphoid tissues such as skin, lung, gut, and liver (14). Comparative transcriptomic analyses pioneered by us and used by other groups, as well as functional studies, have demonstrated the existence of similar mononuclear phagocytes and DC subsets between human and mice (15–20). DC subset candidates have also been described in other mammals such as in ruminants and pigs. However, no systematic study has demonstrated the existence of a framework of homologous DC subsets throughout distant species [for review see Ref. (21)]. Overall, it remains unknown whether a similar diversity in mononuclear phagocyte subsets exists across distant mammals and vertebrates, and when during evolution this complex organization of the mononuclear phagocyte system arose.

The combination of phenotypic, functional, and ontogenic studies used in the mouse model cannot be used to define cell subsets in most other species of interest due to technical, financial, or ethical limitations. As the ontogeny and functions of cell types are instructed by specific gene-expression modules, cell type identity can be defined by its molecular fingerprinting (22). We thus reasoned that mononuclear phagocyte subset identity could be defined by gene-expression profiling, whatever the species. In addition, cell types that are homologous between species must exhibit closer molecular fingerprints and gene-expression programs than non-homologous cell types, based on the definition of homologous cell types as “those cells that evolved from the same precursor cell type in the last common ancestor” (23).

In this paper, we developed a streamlined approach (see Figure S1 in Supplementary Material) to identify homologous mononuclear phagocyte subsets in distant species with reference to the mouse, consisting in (i) designing antibody panels for sorting candidate cell subsets to high marker-based purity, (ii) generating genome expression profiling of the sorted cell subsets, and (iii) performing computational transcriptomic analyses to establish gene signatures and compare them to the transcriptomic fingerprints of the well-characterized immune cell types of the mouse referent species. Our analysis was extended to chicken cell subsets, showing that it is amenable to establish mononuclear phagocyte subset homology throughout vertebrates. We also derived gene-expression signatures and gene interaction networks that are selectively expressed in mononuclear phagocyte subsets in a conserved manner throughout distant mammals and that can be used to identify homologous subsets throughout species. The conserved gene-expression signatures and networks not only encompassed genes with known functions in mononuclear phagocyte subsets but also pointed out novel candidate genes likely involved in the ontogeny or functional specialization of these cell types. Finally, we conducted a phylogenetic analysis to examine the presence in bony fishes and in Lamprey of orthologs of genes from the transcriptomic signatures identified in mammals.

## Materials and Methods

### Pigs and sheep for blood collection

All animal experiments were carried out under licenses issued by the Direction of the Veterinary Services of Versailles (accreditation numbers B78-93) and under approval of the Committee on the Ethics of Animal Experiments of AgroParisTech and INRA-Jouy-en-Josas (COMETHEA, authorization number 00604.01). The eight pigs (blood) used in this study (four males, four females) were around 2 years old and weighted between 60 and 85 kg. Down-sized pigs were kept at the Centre d’Imagerie Interventionnelle (Jouy-en-Josas). «Prealpe» female sheep (total 37, 50–80 kg), originate from and were raised in the «Unité Commune d’Expérimentation Animale» in Jouy-en-Josas, France. Blood (<400 ml/animal) was collected by venous puncture on sodium citrate.

### Isolation of DC subset candidates, B lymphocytes, and Mo from pig blood

PBMC were obtained from pig peripheral blood buffy coat samples by 1.076 g/ml density Percoll (GE Healthcare) gradient centrifugation (24). For B cell sorting, PBMC were surface-labeled with 2 μg/ml primary monoclonal antibody (mAb) against IgL (K139 3E1, IgG2a) followed by Alexa647-conjugated goat anti-mouse IgG2a antibodies (Invitrogen). For pDC sorting, PBMC were surface-labeled with 2 μg/ml primary mAb anti-pig CD4 (PT90A, IgG2a), CD3 (8E6, IgG1), CD14 (CAM36, IgG1), and CD172A (74-22-15, IgG2b) followed by Alexa488, phycoerythrin (PE), or Alexa647-conjugated goat anti-mouse isotype-specific antibodies (Invitrogen). Blood pDC candidates were sorted as CD3^−^ CD14^−^ CD4^+^ CD172^int^ cells, based on previously published indicative data (25). For cDC candidates and Mo sorting, PBMC were surface-labeled with 2 μg/ml mAb anti-pig IgL (K139 3E1, IgG2a), anti-pig IgG (K138 4C2, IgM), anti-pig IgM (PG145A, IgM), anti-pig CD4 (PT90A, IgG2a), anti-human and pig cross-reacting CD14 (TUK4, IgG2a), anti-pig CD172A (74-22-15, IgG1), anti-artiodactyl MHC class II (Th21A, IgG2b), and chicken anti-human and artiodactyl cross-reacting CADM1 (3E1, IgY). The primary antibodies were revealed with Alexa488, PE, or Alexa647-conjugated goat anti-mouse isotype-specific antibodies and with donkey anti-chicken IgY Peridinin Chlorophyll Protein Complex (PerCP)-conjugated IgG. The cDC2 candidates were isolated as FSC^hi^ IgL^−^ IgG^−^ IgM^−^ CD4^−^ CD14^−^ MHC class II^+^ CADM1^−^ CD172^hi^ or CD172^int^ cells. The cDC1 candidates were isolated as FSC^hi^ IgL^−^ IgG^−^ IgM^−^ CD4^−^ CD14^−^ MHC class II^+^ CADM1^+^ CD172^lo^ cells. Mo candidates were sorted as MHC class II^−^ CD172^hi^ cells. Non-relevant antibodies (IgG1, IgG2a, IgG2b, and IgM) were systematically used as controls to measure the level of non-specific background signal caused by primary antibodies. The cell subsets were sorted by flow cytometry on the ImaGif Cytometry platform using the analyzer-sorter MoFlo XDP cytometer and the Summit 5.2 software from Beckman Coulter (cytometric assessment of post-sort purity >98%). The numbers of DCs that were collected per pig lay between 2 and 3 × 10^5^ for pDC, 25 and 47 × 10^3^ for cDC1, 20 and 40 × 10^5^ for cDC2 candidates.

### Isolation of DC subset candidates from sheep blood and B lymphocytes and macrophages from sheep spleen

Sheep PBMC were loaded on 1.065 density iodixanol gradient (Optiprep, Nycomed Pharma) to isolate low density cells from blood. Sheep pDC candidates were isolated by flow cytometry as previously described (26). For isolating sheep cDC candidates, the low density PBMC from several sheep were reacted with anti-CD11c mAb (2 μg/ml, OM1 clone, IgG1) followed by a saturating concentration of pacific blue-labeled anti-mouse IgG donkey Fab (50 μg/ml). After extensive wash, cells were further incubated anti-CD172A mAb (2 μg/ml, ILA24, IgG1) followed by a saturating concentration of Alexa488-labeled anti-mouse IgG donkey Fab (50 μg/ml). After extensive wash, cells were incubated with 2 μg/ml primary mAbs anti-ruminant B cells (DU-204, IgM), CD11b (ILA130, IgG2a), TCR1γ/δ receptor (CC15, IgG2a), CD45RB (CC76, IgG1), and chicken anti-human and artiodactyl cross-reacting CADM1 (3E1, IgY). The IgM and IgG2a primary antibodies were revealed with PE-conjugated goat anti-mouse isotype-specific antibodies, the IgG1 anti-CD45RB primary antibody was revealed with Alexa647-conjugated goat anti-mouse IgG1 antibody, and the anti-CADM1 with anti-IgY PerCP-conjugated IgG. The cDC2 candidates were isolated by flow cytometry as B^−^ CD11b^−^ TCR1^−^ CD45RB^−^ CD11c^+^ CADM1^lo^ CD172^hi^ FSC^hi^ cells. The cDC1 candidates were isolated by flow cytometry as B^−^ CD11b^−^ TCR1^−^ CD45RB^−^ CD11c^+^ CADM1^hi^ CD172^lo^ FSC^hi^ cells. The numbers of DCs that were collected per sheep lay between 1 and 2 × 10^5^ for pDC, around 600 for cDC1, and around 4000 for cDC2. The far lower amounts of collected blood cDCs from sheep as compared to pig may probably originate from the multiple staining steps due to the necessity to separately identify several IgG1 as primary antibodies. B cells and MP were sorted by flow cytometry from isolated sheep spleen cells using the anti-ruminant B cell (DU-204, IgM) and anti-CD14 (CAM36, IgG1), respectively.

### Production of sheep MoDC

Three independent cultures of sheep MoDC were produced with GM-CSF as previously described (27).

### RNA extraction and hybridization on microarrays

Total RNA from subsets was extracted using the Arcturus PicoPure RNA Isolation Kit (Arcturus Life Technologies) and checked for quality with an Agilent 2100 Bioanalyzer using RNA 6000 Nano or Pico Kits (Agilent Technologies). All RNA samples had an RNA integrity number (RIN) above 8.5. When insufficient total RNA amounts for hydridization were obtained (<25 ng for sheep DNA chips, <50 ng for pig DNA chips), the RNAs from the sorted subsets of distinct animal were mixed. RNA amplification and labeling was performed using the one-color Low Input Quick Amp Labeling kit (Agilent Technologies) following the manufacturer’s recommendations. Each RNA sample (25 ng for sheep and 50 ng for pig) was amplified and cyanin 3 (Cy3) labeled, and subsequently the complementary RNA (cRNA) was checked for quality on a Nanodrop and on an Agilent 2100 Bioanalyzer. The cRNAs (600 ng) were fragmented and used for hybridization on custom-designed Agilent ovine and porcine arrays. Our arrays for sheep and pig were custom-designed based on the commercial ovine Agilent arrays for these two species, as previously described (28, 29). In brief, the commercial probes with poor Sigreannot scores (30) were replaced with new probes designed using the e-array software from Agilent Technologies and including ovine or porcine orthologs of genes known to be selectively expressed in human and mouse DC subsets (15). After hybridization of the cRNAs on the custom-designed ovine array, the chips were washed according to the manufacturer’s protocol and scanned using a G2565CA scanner (Agilent Technologies) at the resolution of 3 μm. The resulting .tiff images were extracted using the Feature Extraction software v10.7.3.1 (Agilent Technologies), using the GE1_107_Sep09 protocol. All the protocols used can be obtained by contacting the CRB GADIE facility^1^. The transcriptomic data from the chicken immune subsets were obtained from a previous study (31). All microarray data have been deposited in the Gene Expression Omnibus (GEO) database under reference numbers GSE9810, GSE53500, GSE55642 which have already been released and GSE66311 which is under embargo until publication of the present study.

### Computational pipeline to assess cell subset homology across species

We have designed a computational pipeline in order to define cell subset homology across species, based on the analysis of gene-expression microarray data. In the current study, it has been applied to identify homology relationships between mononuclear phagocyte cells in mammalian species and then extended to the comparison with a more distant species (chicken). However, it can be applied to any cell type and to any species, provided that the annotations of the genes for each species are sufficiently well documented to allow the retrieval of the orthologous genes. In order to perform the comparison of expression profiles of cells coming from different species, thus from different platforms, we have designed two independent procedures. The first procedure (Figure S2A in Supplementary Material) is based on the assessment of the conservation of cell-specific fingerprints/signatures, as assessed by performing gene set enrichment analyses (GSEA, see below) between pairs of cell types. The second procedure (Figure S2B in Supplementary Material, see below) consists in cross-normalizing the expression datasets coming from the different species, in order to simultaneously examine the relationships between all cell types together.

### Cross-species transcriptome comparison by pairwise gene set enrichment analyses

The methodological pipeline is depicted in Figure S2A in Supplementary Material, based on an example with comparison of three different species (A, B, and C). Species A is the reference species, i.e., the species for which the cell types are the most accurately described and generally also for which gene orthologous relationships can be retrieved from (mouse or human here). Species B and C are the test species, i.e., the species for which the identity of the cell types has to be established. Coming from three different platforms, the expression datasets have different numbers of probes, illustrated by boxes of different size. In brief, the strategy is to examine by GSEA whether the transcriptomic fingerprint of a given cell type (X) from the referent species A is enriched in one cell type (Y) of a test species (B for example) as compared to all other cell types of the same species. If this is the case, this would support the hypothesis that the cell type Y from the test species B is homologous to the cell type X of the referent species A. To perform these high-throughput GSEA in a processive way that could be easily reproduced and interpreted by other researchers devoid of bio-informatics expertise, we designed and implemented a dedicated software, called Bubble GUM (manuscript in preparation in which an extensive description of the software will be provided)^2^. Bubble GUM encompasses two main modules, GeneSign and BubbleMap, respectively, dedicated to the generation of gene sets and to their use for GSEA applied to multiple pairwise comparisons of samples integrated together into a simple graphical output that helps in the interpretation of the results. The first step consists in extracting from the reference species the transcriptomic fingerprints of each cell type. A cell-specific transcriptomic fingerprint can be defined as the list of genes that are more highly expressed in the cell type of interest than in all other cell types. These fingerprints were extracted using the “Min (test) vs. Max (ref)” method [(minimum expression among all replicates for all samples for which the transcriptomic fingerprint is defined/maximum expression among all replicates used as reference) ≥1.5-fold] (15, 32), using the GeneSign module of Bubble GUM. These transcriptomic fingerprints, in gene symbol format, will be assessed for enrichment on the expression datasets of species B and C. Thus, it is necessary to convert the probe annotations from the arrays of species B and C into the gene symbol of their orthologous counterparts in species A. For this purpose, we used the orthology relationships defined by the Sigenae pipeline which annotated the pig and sheep genes with their human and mouse orthologous gene symbols (30). The genes present on the gene chips of species B and C that were not associated to an orthologous counterpart in species A remained annotated with the gene symbol corresponding to their species of origin. The statistical enrichment of the cell-specific transcriptomic fingerprints extracted from the reference species A were then calculated between pairwise comparisons of cell types from species B or C with the GSEA methodology, using gene set permutations for computing the *p*-values and false-discovery rates (FDR) (33). This was achieved, and the results graphically represented, by using the BubbleMap module of Bubble GUM.

### Cross-normalization of the species-specific expression datasets

Using the same starting expression datasets, this is an alternative strategy which is complementary to the pairwise GSEA of the species-specific expression datasets, since it allows clustering all cell types together based on the overall evaluation of the proximity of their expression patterns of hundreds to thousands of orthologous genes. The first step (Orthology Filter) consists in aligning the genes across the species (A, B, and C). It requires retaining only one representative probe per gene for each species/platform. This is needed since, in microarray designs, many genes are often each represented several times by a number of individual probes having each a different signal-to-noise ratio. However, probes have no equivalence across species, whereas genes do. In our experience, the signal-to-noise ratio is generally better for probes that have the strongest signal in positive control samples, while certain probes that have a low signal-to-noise ratio can give misleading high fold changes across conditions when using a limited number of replicates. Hence, we computed for each probe in each platform the sum of normalized expression values across all samples and kept for each gene the probe that had the highest computed value. Then, for the genes of species B and C, we retrieved the gene symbol of their orthologous counterparts in species A (reference species). In the example illustrated in Figure S2B in Supplementary Material, species A is the reference: the genes of species B and C are thus annotated using the gene symbol of the orthologous genes in the species A. The genes not represented in each of the gene chip platform used were removed from the analysis. This Orthology Filter yielded a filtered expression dataset for each species, where the number of genes and their associated symbols were similar between all species, as illustrated by boxes of the same size (Figure S2B in Supplementary Material). In order to be able to rigorously merge the different datasets together, the dynamic ranges of expression values for each gene across all species must be homogenized by setting for each dataset and for each gene across all samples the mean expression to 0 and the variance to 1, a process called data centering and reduction. To prevent this mathematical transformation of the expression data to introduce noise by forcing artifactual expression changes for genes that were unregulated in the initial datasets, it is mandatory to remove all the genes that are not regulated in at least one of the datasets. This thus requires keeping only the genes that are differentially expressed between at least two cell types for each of the species studied. This was achieved in the second and third steps of the data processing. The second step (Differentially Expressed Gene, DEG, Filter) consisted in identifying independently in each dataset the genes that are differentially expressed between at least two cell types. The identification of DEG was performed by calculating the minimal ratio between each pairwise comparison of cell types and by selecting only the genes for which this minimal ratio was higher than twofold. The third step (DEG intersection) consisted in keeping only the genes that were common to all filtered DEG lists, i.e., the orthologous genes which expression was modulated across samples in each of the species studied. The fourth step consisted in data centering and reduction for each dataset, which was performed using the R statistical environment. This step consists in setting, for each dataset, the mean to 0 and the variance to 1, so that all datasets are comparable. In the fifth and final step, the different datasets were merged together simply by aligning their rows based on the common gene symbol extracted from species A. The final cross-normalized expression dataset including the data for all species was then used to perform canonical analyses for classification of samples, namely here hierarchical clustering.

### Generation of conserved cross-species cell type-specific signatures

For each species (human, mouse, sheep, and pig), the transcriptomic fingerprint of each cell type was generated by selecting the genes more highly expressed in the cell type of interest, as compared to all other studied cell types of the same species in the case of “absolute” transcriptomic signatures, or as compared to selected cell populations of the same species in the case of “relative” transcriptomic signatures, using the “Min (test) vs. Max (ref) ≥1-fold” method. Once the fingerprints had been obtained for each species for a given cell type, the gene identifiers were all converted into their corresponding official human gene symbol using BioMart and we selected the intersection of these four lists as the final conserved cross-species transcriptomic signature specific of that cell type, with the following exceptions. First, for certain cell types such as MoDC, data were available from only three, and not four, species. Second, in order to avoid removing putatively relevant signature genes, we kept in these signatures the genes found in all species but one, when their absence in the signature of that given species was due to absence or non-functionality of corresponding ProbeSets on the array of that species.

### Real-time PCR

For relative quantitation of gene expression in subsets, RNA was reverse transcribed using random primers and the Multiscribe reverse transcriptase (Applied Biosystems). Real-time PCR (qPCR) was carried out with 300 nM primers in a final reaction volume of 25 μl of 1 X SYBR Green PCR Master Mix (Applied Biosystems). The primers used to amplify ovine and porcine cDNA were designed with the Primer Express software (v2.0) using publically available GenBank sequences (Table S1 in Supplementary Material). PCR cycling conditions were 95°C for 10 min, linked to 40 cycles of 95°C for 15 s and 60°C for 1 min. Real-time qPCR data were collected by the Mastercycler^®^ e0p realplex-Eppendorf system and 2^−ΔCt^ calculations for the relative expression of the different genes (arbitrary units) were performed with the Realplex software using GAPDH for normalization. All qPCR reactions showed >95% efficacy.

## Results

### Isolation of mononuclear phagocyte subset candidates from artiodactyl blood or spleen using a set of surface markers

**Figure 1:**
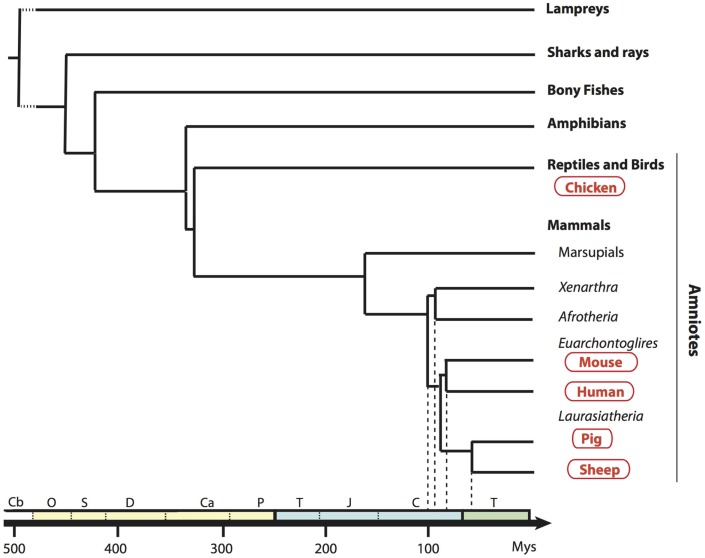
**Phylogenetic tree of a broad selection of vertebrates**. Phylogenic relationships and divergence time of clades are drawn according to Hajjoubi et al. (45), de Jong et al. (46), and Douady et al. (47). Geological periods are indicated at the bottom in the following order: primary era in yellow with Cambrian (Cb), Ordovician (O), Silurian (S), Devonian (D), Carboniferous (Ca), Permian (P); secondary era in blue with Trias (T), Jurassic (J), Cretacean (C), and the tertiary era (T) in green.

**Figure 2:**
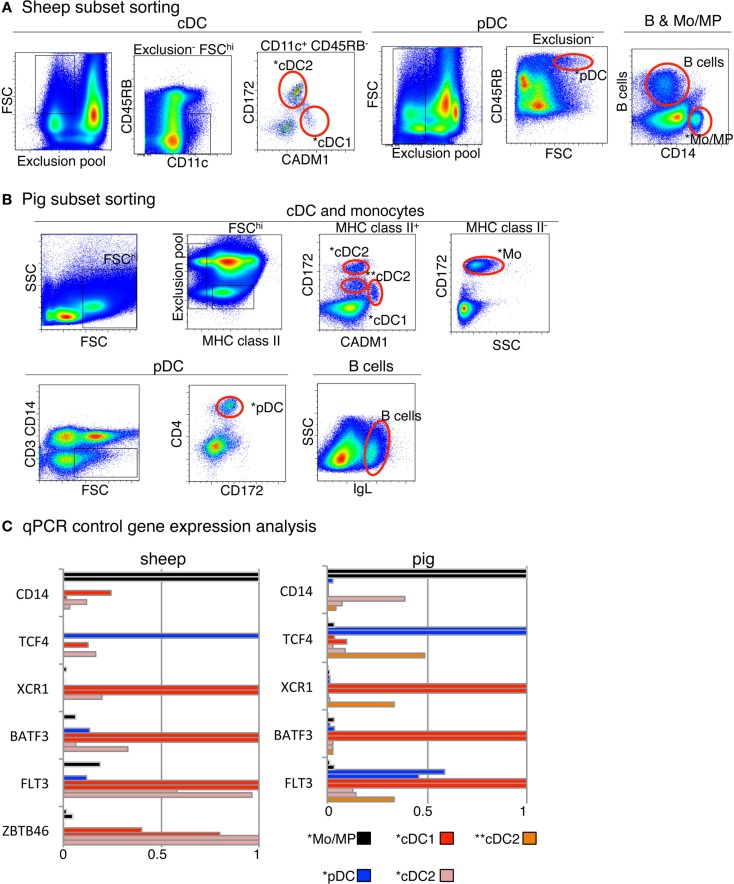
**Sorting of B cells, DC subset candidates, and Mo/MP candidates from pig and sheep blood or spleen and analysis of their expression of control genes**. **(A)** Sheep cell subset sorting from blood and spleen. For sorting of blood cDC subset candidates, low density blood cells were gated on FSC^hi^ CD11c^+^ B^−^ CD11b^−^ TCR1^−^ CD45RB^−^ cells and analyzed for CADM1 and CD172 expression, based on isotype control references for each staining. The CADM1^hi^ CD172^lo^ (*cDC1) and CADM1^lo^ CD172^+^ (*cDC2) cells were sorted. Blood pDC candidates (*pDC) were sorted as low density FSC^hi^ B^−^ CD11b^−^ TCR1^−^ CD8^−^ CD11c^−^ CD45RB^+^ cells. Splenic candidate *Mo/MP were sorted as CD14^+^ cells. Splenic B cells were identified as DU-2-104^+^ cells. **(B)** Pig cell subset sorting from blood. For cDC candidate sorting, low density PBMC were gated on FSC^hi^ MHC class II^+^ B^−^ CD14^−^ CD4^−^ cells and analyzed for CADM1 and CD172 expression. One cDC1 candidate population was identified and sorted, as CADM1^+^ CD172^lo^ (*cDC1). Two cDC2 candidate populations were identified and sorted, as CADM1^−^ CD172^hi^ (*cDC2) and CADM1^−^ CD172^int^ (**cDC2). Candidate Mo were sorted as CD172^+^ MHC2^−^ cells (*Mo). Candidate pDC were sorted as CD3^−^ CD14^−^ CD4^+^ CD172^int^ cells (*pDC). B cells were identified and sorted as IgL^+^ cells. **(C)** qPCR analysis of the expression of control genes in sorted candidates from one or two animals. RNA from candidate cell subsets (left, sheep; right, pig) were subjected to detection of control transcripts by qPCR. Control transcripts were chosen based on their high selective expression in specific subsets of mononuclear phagocytes in a conserved manner between mouse and man, i.e., *TCF4* for pDC, *FLT3*, *BATF3* and *ZBTB46* for cDC, *XCR1* for cDC1, and CD14 for Mo/MP. Data are represented as relative expression levels normalized to maximal expression across cell types, each bar corresponding to a distinct animal.

In order to establish a framework of homologous mononuclear phagocyte subsets across different species, we selected two mammalian artiodactyl species, sheep and pig, belonging to the Laurasatherians, a phylogenetically distant order from the Euarchontoglires that include the human and mouse species (Figure 1). A set of available antibodies exist to isolate cell subsets in these species of interest as food animals, hosts of zoonotic diseases, and biomedical models. We focused on blood or spleen immune subsets, because (i) a large source of transcriptomic data is available from this compartment in the human and mouse reference species, (ii) they are readily accessible with a minimum of technical biases in all species, and (iii) their gene-expression profiles are not expected to be influenced by peripheral tissue imprinting. We designed antibody panels to sort the subsets. In human and mice, cDC lack expression of T and B lymphocyte and Mo/MP markers and they abundantly express CD11c and MHC class II. Independent groups identified SIRPα as a conserved marker suitable to distinguish cDC2 from cDC1 across species (17, 34). Whereas XCR1 stands as the best marker for identifying cDC1 (34–41), appropriate reagents are not yet available in species outside human and mouse, and CADM1, whose sequence is highly conserved in evolution (42), can be used as an alternative (43, 44). cDC1 and cDC2 candidates were isolated from sheep and pig low density blood cells after exclusion of irrelevant cells (Figure 2A for sheep and Figure 2B for pigs, see Material and Methods section). The «candidate» nature of a sorted cell subset is marked by a star before the considered subset name in this paper. Due to restricted reagent availability, CD11c and MHC2 class II markers were used to isolate sheep and pig cDC, respectively.

In the case of pig, two populations being CADM1^−^ CD172^+^ or CD172^int^ were identified and selected as potential candidates and designated as *cDC2 and **cDC2, respectively (Figure 2B). We previously published the marker phenotype, morphology, and type I IFN production properties of sheep lymph and blood *pDC as CD45RB^+^ FSC^high^ TCRγ/δ^−^ B^−^ CD11b^−^ cells (26, 48). The sorted cells were very potent at type I IFN production upon viral-type stimulation, demonstrating at the functional level that they were highly enriched in pDC. Moreover, the sorted cells had the expected size and plasmacytoid morphology, indicating that they were not contaminated by other types of myeloid cells (48). Blood *pDC from pigs were sorted as CD3^−^ CD4^+^ CD172^dim^ cells based on marker phenotype, morphology, and type I IFN production properties established by others (25, 49). Pig *Mo were sorted as CD172^high^ MHC class II^−^ cells and sheep splenic *MP as CD14^+^ cells.

To decrease the risk of improper identification of sorted cell subsets, we performed a quality control consisting in examining the expression of a few control genes by qRT-PCR (Figure 2C) prior to performing genome-wide transcriptomic analyses. Control genes were chosen based on their high selective expression in a given subset of mononuclear phagocytes in a conserved manner between mouse and human (15, 36) and encompassed *TCF4* for pDC, *CD14* for Mo/MP, *FLT3* for cDC and pDC, *ZBTB46* for cDC, *BATF3* and *XCR1* for cDC1. As expected, *TCF4* was expressed to much higher levels in sheep (26) and pig *pDC as compared to all other cell types examined except for pig **cDC2. *CD14* was expressed at much higher levels in sheep and pig *mono/MP as compared to all other cell types examined except one of the two replicates of pig *cDC2. *FLT3* was expressed at much higher levels in sheep and pig *cDC1 and in sheep *cDC2 as compared to all other cell types examined. *BATF3* and *XCR1* were expressed at higher levels in sheep and pig *cDC1 as compared to all other cell types examined. Importantly, these control analyses have allowed us to improve our initial strategy for sheep *cDC1 and *cDC2 sorting. In fact, in our initial sorting (Figure S3 in Supplementary Material), the CD45RB^+^ cells were not excluded to sort cDC candidates, and the *cDC1 were found to express high levels of *TCF4* mRNA, leading us to refine the sheep cDC sorting as presented in Figure 2. Thus, overall these control analyses validated our strategy for phenotypic identification and flow cytometry purification of sheep and pig *pDC, *Mo/MP and *cDC1 DC, and of sheep *cDC2. In the case of pig cell subsets, the nature of **cDC2 and *cDC2 was not clear since the former expressed high levels of *TCF4* and *XCR1*, and the latter expressed relatively high levels of *CD14* in one out of two replicates. Because pig **cDC2 presented a relatively high expression level of both *TCF4* and *XCR1*, we concluded that they were significantly contaminated by pDC and cDC1. Therefore, we excluded these cells from further analyses and assumed that pig *cDC2 cells were the proper candidate.

### Use of pairwise gene set enrichment analyses for assessment of the similarity between mononuclear phagocyte subsets across distant mammal species

**Figure 3:**
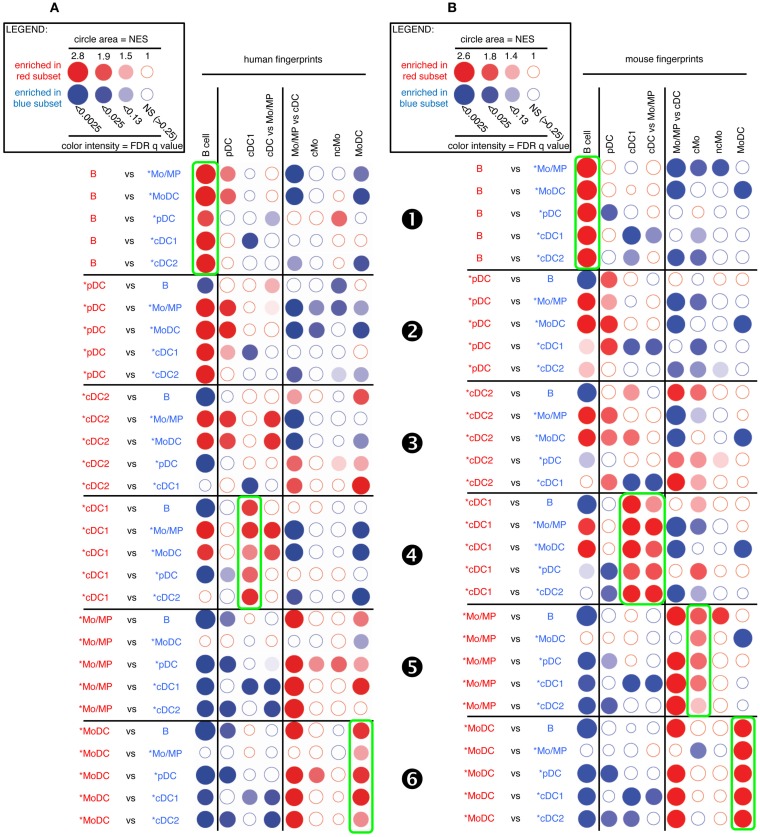
**GSEA-based assessment of the identity of sheep cell subset candidates by comparison with well-defined human and mouse mononuclear phagocytes**. Candidate sheep cell subsets were compared to one another for their relative enrichment in transcriptomic fingerprints (GeneSets) specific of human **(A)** or mouse **(B)** mononuclear phagocyte subsets, using GSEA through the Bubble GUM software. The human and mouse GeneSets were defined through the same approach based on pre-existing knowledge of equivalency between human and mouse mononuclear phagocytes. A GeneSet specific for B cells was included as a control for the methodology, since the identity of this cell type is clearly established in all species and its homology across species is undisputed. The GeneSets used were named and defined as follows. The transcriptomic fingerprints “B cell,” “pDC,” “cDC1,” “cMo,” “ncMo,” and “MoDC” consisted in the lists of human/mouse genes showing a high selective expression in the eponym human/mouse cell subset as compared to many other leukocytes (see Materials and Methods for further details, Data Sheet S2 in Supplementary Material). The transcriptomic fingerprints “cDC vs. Mo/MP” and “Mo/MP vs. cDC” consisted in the lists of human/mouse genes expressed in cDC to higher levels than in Mo/MP, and reciprocally (Data Sheet S2 in Supplementary Material). All possible pairwise comparisons between sheep cell subsets were performed to assess their respective expression of the transcriptomic fingerprints of human and mouse mononuclear phagocyte subsets, using the Bubble GUM software for calculations and graphical output. Results are represented as bubbles, in a color matching that of the cell subset in which the GeneSet was enriched. Stronger and more significant enrichments are represented by bigger and darker bubbles, as illustrated in the legend box of the figure. Specifically, the surface area of bubbles is proportional to the absolute value of the normalized enrichment score (NES). The color intensity of dots is indicative of the false-discovery rate (FDR) statistical value.

**Figure 4:**
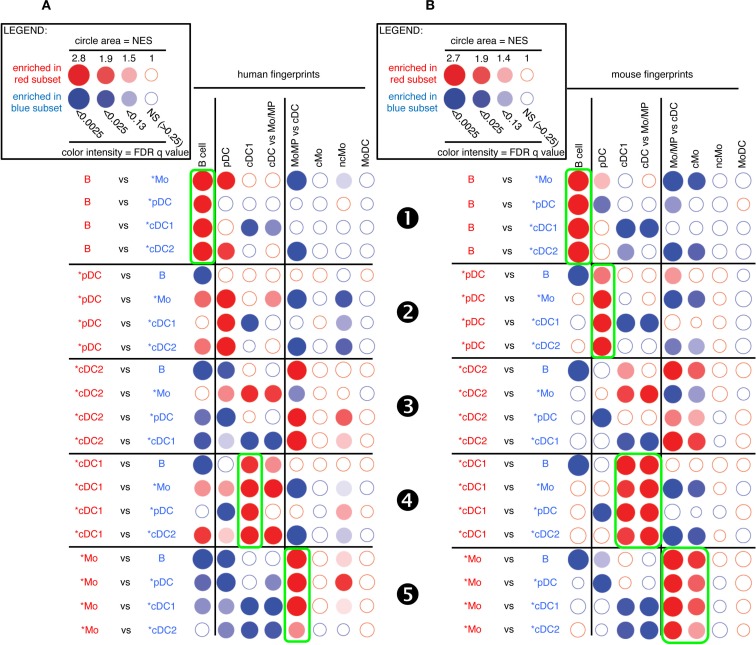
**GSEA-based assessment of the identity of pig cell subset candidates by comparison with well-defined human and mouse mononuclear phagocytes**. The gene-expression data for pig cell subset candidates were analyzed exactly as described in Figure 3 for sheep cell subset candidates. Candidate swine cell subsets were compared to one another for their relative enrichment in transcriptomic fingerprints specific of human **(A)** or mouse **(B)** mononuclear phagocyte subsets, using GSEA through the Bubble GUM software.

As a first approach to establish mononuclear phagocyte subset homology across species, we determined the level of similarity between artiodactyl, mouse, and human mononuclear phagocytes using pairwise GSEA, as previously performed to characterize human immune cell subsets (19) and chicken cDC (31). To that aim, we used publicly available transcriptomic data from a selection of human and mouse immune cell types (Data Sheet S1 in Supplementary Material). We established human and mouse transcriptomic fingerprints for B cells, pDC, cDC1, Mo/MP, MoDC, cMo, and ncMo as the list of genes that are expressed at least 1.5-fold higher in the index cell population than in a large number of other immune cell types (Data Sheet S2 in Supplementary Material). B lymphocytes were chosen in all species as a reference cell subset, because their phenotypic identification in each species and their homology across species are already well established, and because they are expected to share with mononuclear phagocytes a genetic program underlying their common function of antigen-presenting cells. We generated a common fingerprint for Mo and tissue MP because their gene program is very close in the mouse (9), even though tissue MP generally derive from embryonic precursors rather than from circulating blood Mo. We could not establish a human or mouse cDC2 transcriptomic fingerprint with a sufficiently large number of genes for subsequent reliable statistical analysis. We also defined relative transcriptomic signatures for cDC vs. Mo/MP as the list of genes that are 1.5-fold higher in all cDC relatively to Mo and MP from different tissues, and reciprocally (Data Sheet S2 in Supplementary Material). Finally, we identified transcriptomic fingerprints from human and mouse MoDC (12). We then tested whether the transcriptomic signatures of mouse and human immune cell types were enriched between sheep or pig candidate cell subsets using GSEA (33) (Figures 3 and 4). As control, since homologies between mouse and human cell subsets have been previously demonstrated by other methods of transcriptional analyses (15, 16, 18), mouse fingerprints were also used for GSEA analysis on human cells (Figure S4 in Supplementary Material) and reciprocally (Figure S5 in Supplementary Material).

As expected, sheep B cells were significantly enriched for the expression of both human and mouse B cell transcriptomic fingerprints as compared to all other sheep cell subsets examined (Figure 3, ❶). The sheep *pDC were enriched for the human and mouse pDC fingerprints in most comparisons (Figure 3, ❷), suggesting that sheep *pDC correspond to homologs of human and mouse pDC. However, both mouse and human pDC fingerprints were not significantly enriched in the comparison of sheep *pDC with *cDC2 (NES = 1.29 and 1.24, and FDR = 1.0 and 1.0, respectively), indicating that sheep pDC probably contaminate sheep *cDC2 despite exclusion of CD45RB^+^ cells for their purification. The sheep *pDC were also enriched for the human B cell fingerprint in most comparisons (except with sheep B cells), what can be partly explained by the known overlap between the gene-expression program of pDC and B (15, 50–52); however, the human pDC fingerprint is not enriched in the sheep *pDC comparison with B cells and the extent of the human B cell fingerprint enrichment in sheep *pDC is above the expectations provided by similar analyses in the human and mouse reference species (Figures S4 and S5 in Supplementary Material), all of this indicating that B cells are likely to contaminate sheep *pDC despite exclusion with a pan-B cell marker for *pDC selection. Finally, *cDC2 did not show a clear enrichment for any human and mouse signatures (Figure 3, ❸). However, it is also the case when examining enrichment of mouse cell subset fingerprints in human cDC2 (Figure S4 in Supplementary Material, ❸) and reciprocally (Figure S5 in Supplementary Material, ❸). Hence, this GSEA approach is not very informative for identification of cDC2, due to the lack of robust human or mouse fingerprints that are specific of this cell type as mentioned earlier. Sheep *cDC1 were significantly enriched in the human and mouse cDC1 fingerprints in all comparisons (Figure 3, ❹). They were also enriched systematically in the mouse cDC vs. Mo/MP fingerprints. This suggested that sheep *cDC1 correspond to true homologs to human and mouse cDC1. Sheep splenic *Mo/MP were strongly enriched for the human and mouse Mo/MP vs. cDC fingerprints except when compared to MoDC, and not for the human and mouse fingerprints of B lymphocytes, pDC, or cDC (Figure 3, ❺). This confirmed that sheep splenic *Mo/MP belong to the monocytic lineage and not to the B nor DC lineages. However, their precise identity remained unclear as they were enriched for the mouse cMo fingerprint but not for the human cMo or ncMo fingerprints. When mouse fingerprints were applied on human immune cell subsets comparisons and vice versa, there was also no consistent alignment of ncMo between the two species (Figures S4 and S5 in Supplementary Material, highlights ❺ and ❻). Finally, sheep *MoDC that were derived from bone marrow cells in GM-CSF (27), were systematically and strongly enriched in the human and mouse MoDC signatures (Figure 3, ❻), confirming the homology between these three populations.

A similar analysis for pig candidate cell subsets also clearly established similarities with their putative human and mouse equivalents for B cells (Figure 4, ❶), pDC (Figure 4, ❷), and cDC1 (Figure 4, ❹) but not for cDC2 (Figure 4, ❸). Pig *Mo were clearly enriched for human and mouse fingerprints of cells of the monocytic lineage, and not for human and mouse signatures of B lymphocytes, pDC, or cDC (Figure 4, ❺).

Thus, altogether, GSEA analysis of the sheep and pig data for the fingerprints of human and mouse immune cell subsets gave results as informative as those obtained when comparing together human and mouse cell types, and clearly established similarities between sheep and pig cell subset candidates and their putative human and mouse equivalents for B cells, pDC, cDC1, and MoDC. Further analyses are necessary to precisely identify the nature of sheep and pig *cDC2 and *Mo/MP subsets.

### Confirmation and extension of the conclusions on the similarity between mononuclear phagocyte subsets through global and simultaneous analysis of the gene-expression profiling of all cell types from mammalian species using hierarchical clustering

**Figure 5:**
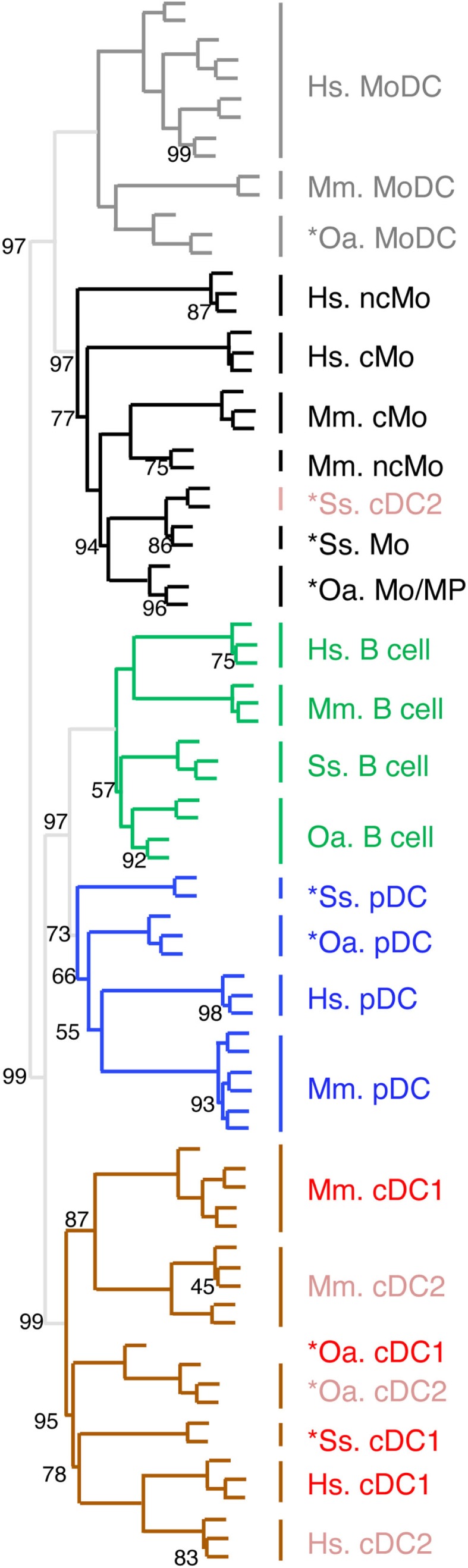
**Confirming and completing homology assignment of sheep and pig candidate Mo/MP, pDC, and cDC by unsupervised hierarchical clustering with human and mouse cell types**. The datasets of each species were filtered and cross-normalized in order to allow mixing them all together for global analysis of the relationships between sheep, pig, mouse, and human mononuclear phagocyte subsets by using unsupervised hierarchical clustering. In brief, this analysis is focused on 1926 unique orthologous genes (i) for which a functional and specific ProbeSets was present on the microarrays for each species and (ii) which were found to be differentially expressed in each species between at least two subsets of mononuclear phagocytes. For each species and each of these 1926 genes, the expression data was then transformed to a mean = 0 and a variance = 1, in order to cross-normalize expression values to a similar dynamic range between the different datasets. For each cell type, the initials of the scientific name of the species of origin are indicated as a prefix: Hs, human; Mm, mouse; Ss, pig; and Oa, sheep. The robustness of the tree was tested by multiscale bootstrap resampling using Pearson’s correlation as distance and average linkage as cluster method, with 1000 iterations at 10 different dataset sizes comprised between 50 and 140% of the complete dataset. An AU (approximately unbiased) *p*-value (percentage) was calculated and placed on the nodes of the cluster dendrogram. Missing percentages correspond to 100%.

In order to confirm the identification of homologous mononuclear phagocytes across species as deduced from GSEA analyses, and to potentially gain more insights into the exact nature of pig and sheep *cDC2 and *Mo/MP, we next processed all the data together for global analysis by hierarchical clustering (Figure 5). Only the genes that showed significant variation in their expression across subsets in each species were selected and the resulting datasets were normalized across species. All B cells from the four mammalian species grouped together in a specific branch of the tree, rather than each with other immune cells of the same species. This finding validates hierarchical clustering as an alternative method for identifying homologous mononuclear phagocytes across species. A closer examination of the dendrogram shows that the different cell types grouped in two major branches. The first one encompassed all the known and candidate cells of the monocytic lineages and pig *cDC2, and split further into two subgroups, one including all the identified or candidate MoDC, and the other one including all the identified or candidate Mo/MP and pig *cDC2. The second branch encompassed all the other cell types known or hypothesized not to belong to the monocytic lineage. This branch further split into two sub-branches, one constituted of the group of B cells and of the group of identified or candidate pDC, and the other constituted of identified or candidate cDC subsets except pig *cDC2. The common clustering of B and pDC transcriptome can be explained by the shared gene-expression program between B and pDC as mentioned above. Hence, this analysis confirmed the conclusion already drawn from the GSEA analyses, namely the monocytic nature of sheep and pig *Mo/MP and *MoDC, as well as the homology between pig, sheep, mouse, and human *pDC/pDC. Moreover, the hierarchical clustering analysis allowed to better define the nature of sheep and pig *cDC2. Specifically, it confirmed the hypothesis that sheep *cDC2 belong to the cDC family, while, on the contrary to our *a priori* assignment, it shows that pig *cDC2 rather resemble Mo than cDC. However, within the branch of monocytic cells, this analysis grouped Mo/MP by species of origin rather than by cMo vs. ncMo subsets. Similarly, this analysis grouped cDC by species rather than by cDC1 vs. cDC2 subsets.

### Identification of similarity between subsets of DC and of Mo across species through hierarchical clustering analyses focused on these cell types

**Figure 6:**
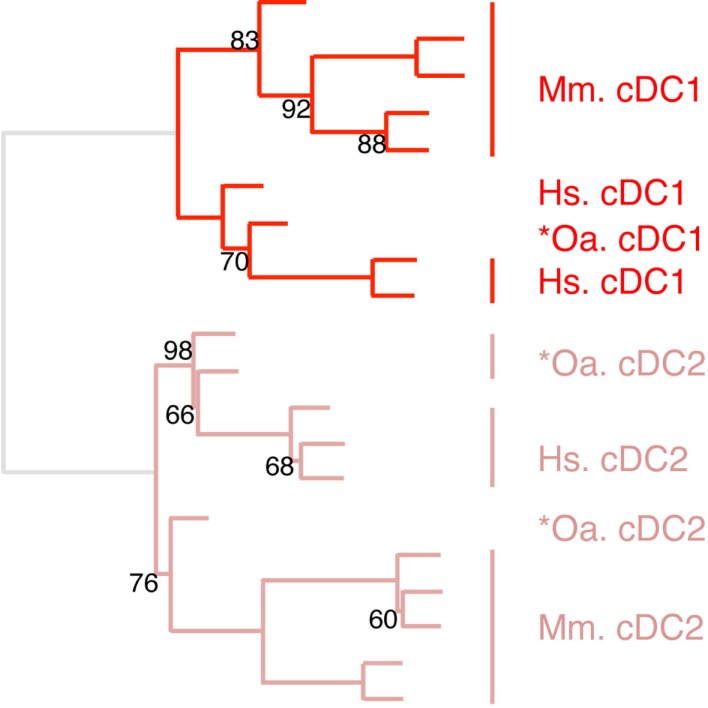
**Confirming homology assignment of sheep cDC1 and cDC2 candidates by unsupervised cross-species hierarchical clustering focused on cDC subsets**. An unsupervised cross-species hierarchical clustering analysis was performed as described in Figure 5, but focused only on cDC subsets. The corresponding filtered dataset included 868 unique orthologous genes found regulated between cDC1 and cDC2 from human (Hs), mouse (Mm), and sheep (Oa). Pig cDC could not be included in this analysis due to the lack of data on proper pig cDC2.

**Figure 7:**
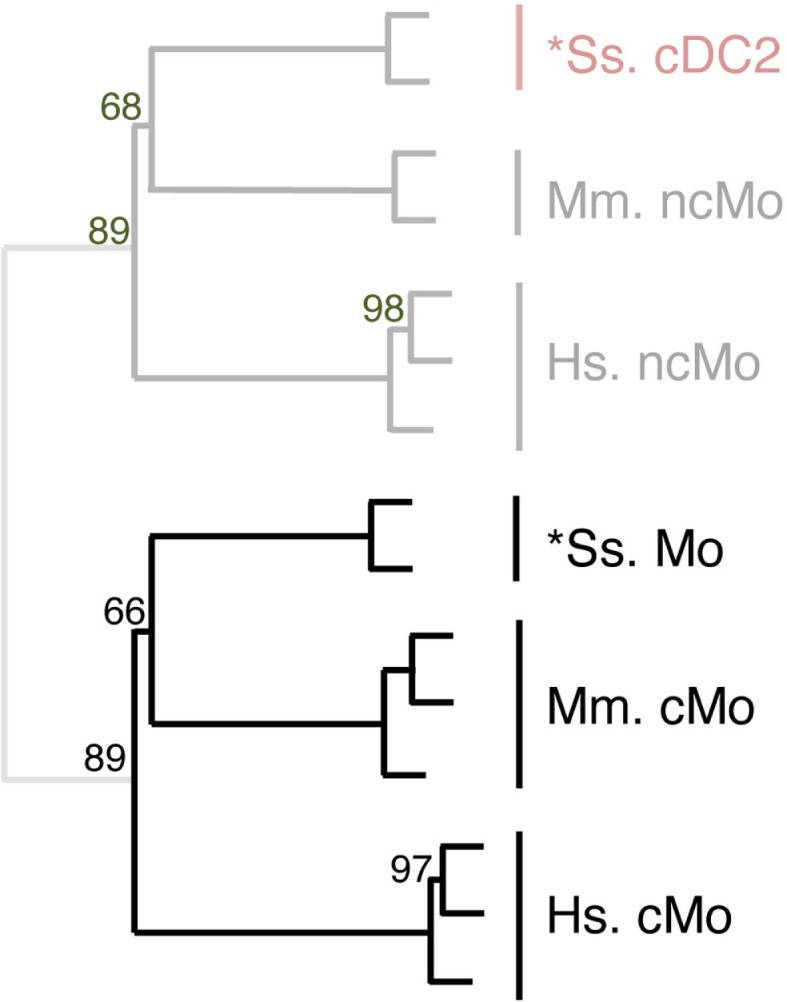
**Completing homology assignment of pig cDC2 DC candidate to non-classical Mo subset by unsupervised cross-species hierarchical clustering focused on Mo subsets**. An unsupervised cross-species hierarchical clustering analysis was performed as described in Figure 5, but focused only on cells from the monocyte branch of the tree obtained in Figure 5. The corresponding filtered dataset included 191 unique orthologous genes found regulated between cMo and ncMo from human (Hs), mouse (Mm), and pig (Ss). Sheep data could not be included in this analysis due to lack of data on subsets of sheep monocytes.

The expression patterns of genes outside of the cell types of interest may mask similarity between cDC or Mo subsets, as previously reported (15). Hence, we further evaluated the similarities between subsets of cDC on the one hand, and of Mo/MP on the other hand, by re-analyzing their gene-expression profiles focusing only on the genes that showed significant variation in their expression across DC subsets (Figure 6) or Mo/MP (Figure 7) in each species. Pig data were not used in the analysis focused on cDC, because, pig *cDC2 belonged to the monocytic branch and not to the DC branch of Figure 5. Sheep data were not used in the analysis focused on Mo/MP, because only one subset of sheep Mo/MP had been purified. Remarkably, these focused analyses grouped samples by cell types rather than by species. The cDC-focused hierarchical clustering confirmed the conclusion drawn from GSEA that sheep, mouse, and human cDC1/*cDC1 are homologs, and refined our understanding of the identity of sheep *cDC2 by showing their homology to mouse and human cDC2 (Figure 6). The Mo/MP-focused hierarchical clustering allowed to newly identify pig homologs to mouse and human cMo vs. ncMo (Figure 7). Pig *cDC2 correspond to ncMo and pig *Mo correspond to cMo. In a complementary phenotypic FACS analysis, we confirmed that likewise human ncMo as compared cMo, pig *cDC2 express higher membrane levels of CD16 and CD163 as compared to pig *Mo (Figure S6 in Supplementary Material).

Altogether, our comparative analyses of the gene-expression profiles of mononuclear phagocyte subsets across mammals indicated that the complex specialization of these cells into distinct subsets is conserved across mammals for both DC and Mo. Subset grouping did not indicate existence of a relationship between transcriptomic proximity of subsets and phylogenetic closeness of species. The conserved organization across distant mammals suggests that the mononuclear phagocyte complexity arose in a common mammalian ancestor and that the different subsets can be considered as homologous subsets across mammals.

### Evidences for homologous cDC and Mo/MP lineages across warm-blooded vertebrates

**Figure 8:**
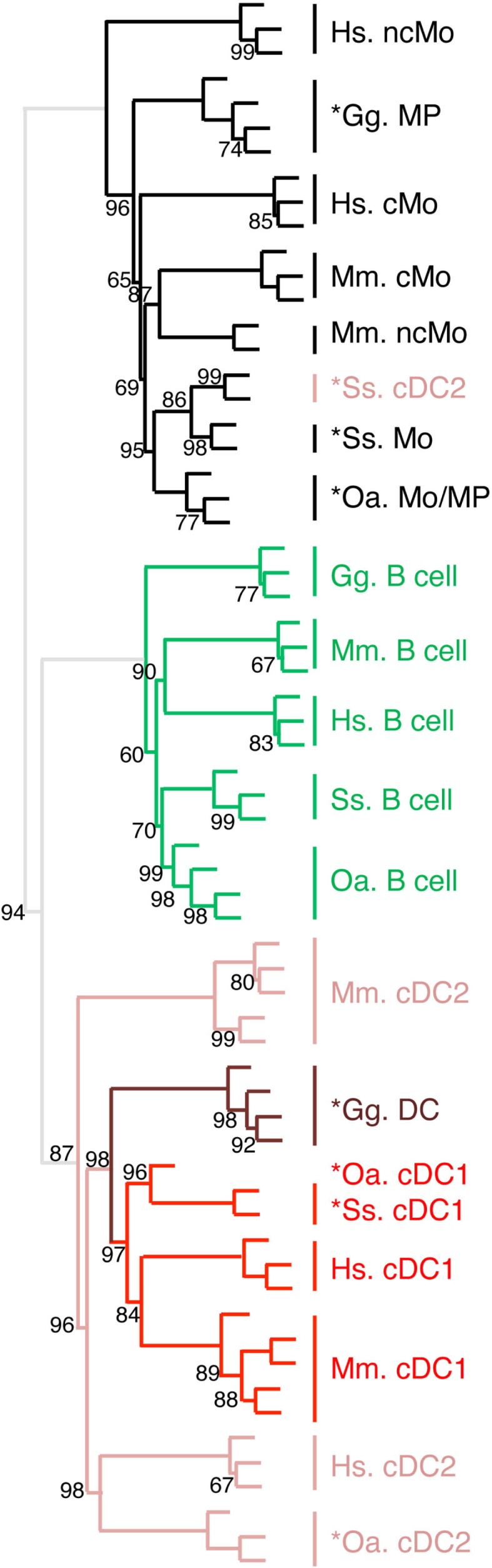
**Unsupervised cross-species hierarchical clustering including a chicken dataset demonstrates a conserved organization of vertebrate mononuclear phagocytes in the two main lineages of Mo/MP vs. cDC**. An unsupervised cross-species hierarchical clustering analysis was performed as described in Figure 5, but including gene-expression data from chicken (Gg prefix for *Gallus gallus*) and focused only on the cell types commonly sorted in all five vertebrate species, i.e., B cells, Mo/MP, and cDC. The corresponding filtered dataset included 388 unique orthologous genes found regulated across cell subsets in each species.

We recently generated the transcriptomic profile of MP, total cDC, and B cells from chicken spleen and found similarities with human and mouse corresponding immune cell subsets by GSEA (31). In order to extend our subset homology analysis to non-mammalian vertebrates, we normalized and processed the transcriptomic data in a hierarchical clustering analysis as described above, using mammalian and chicken Mo/MP, B cells, and cDC subsets (Figure 8). There again, a tree consisting of two main branches was obtained, corresponding to a split between Mo/MP and B cells/DC. In the cDC branch, the cDC1 subset clustered together and included the chicken total cDC. The chicken MP grouped with the mammalian Mo/MP. Whereas this analysis is still partial due to limited knowledge and availability on marker sets for sorting immune cell subsets in chicken, it shows that our transcriptomic comparative approach can be used to define subset homology throughout vertebrates. It also further supports that separation of mononuclear phagocytes into Mo/MP and cDC occurred early during vertebrate evolution and must already have been in place in the common ancestor of reptiles (including birds) and mammals.

### Identification of mononuclear phagocyte gene-expression signatures across mammals

**Figure 9:**
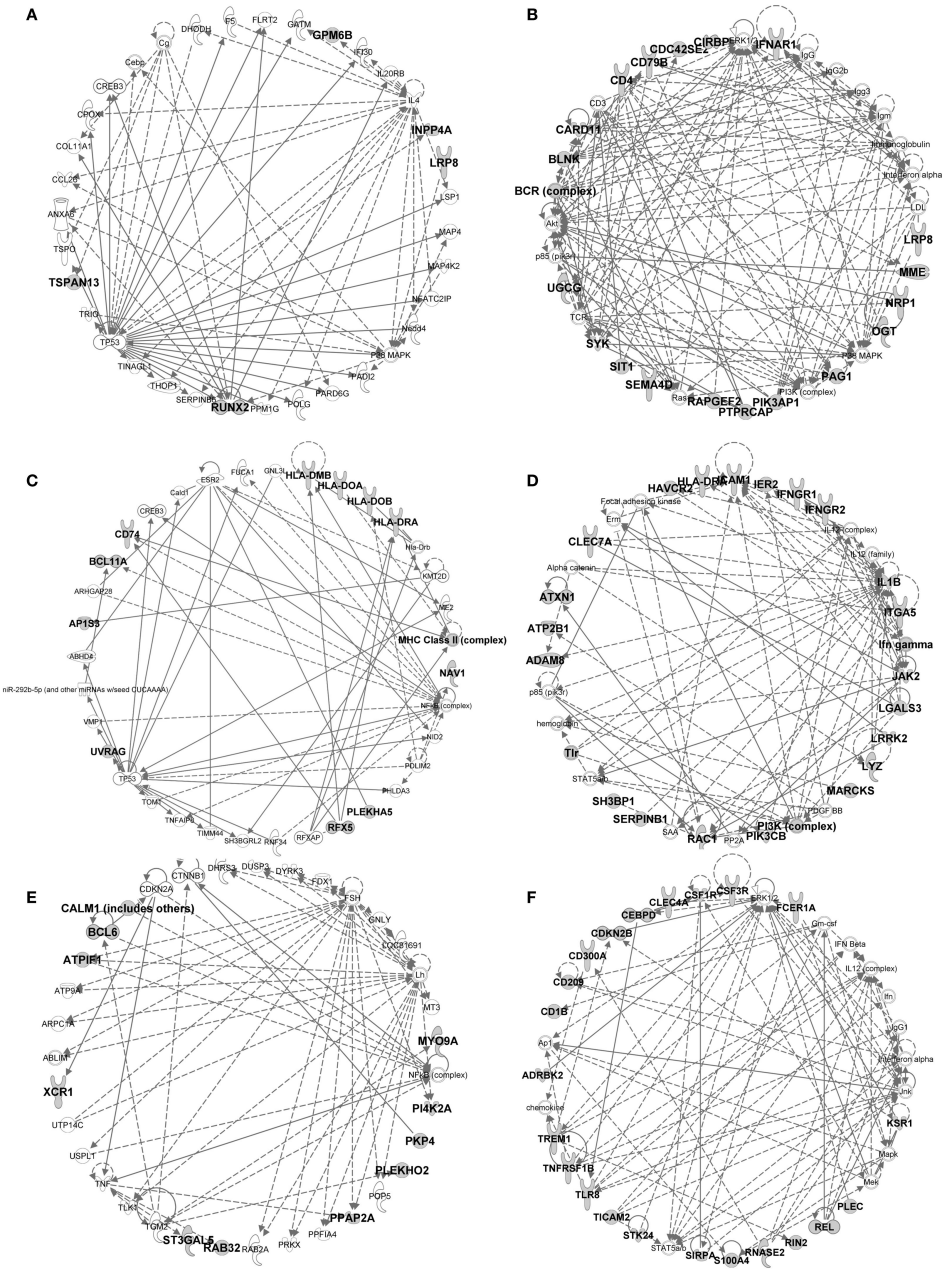
**IPA gene interaction networks of the conserved signatures in subsets of the DC lineage**. The conserved signatures of subsets of the DC lineage were analyzed in Ingenuity Pathway Analysis which generates networks based on the connectivity of the genes in each signature (in boldface) but also on their connectivity with genes not belonging to the signature (in plain characters). The identified networks are displayed as graphs showing the molecular relationships between genes/gene products. Genes are represented as nodes, and the biological relationship between two nodes is represented as an edge (line). The edges can represent direct (continuous) or indirect (dashed) relationships between nodes. Selected networks generated by IPA and covering parts of conserved cell-specific signatures are displayed: **(A)** pDC signature network, **(B)** pDC vs. cDC signature network, **(C)** cDC vs. Mo/MP signature network, **(D)** cDC vs. pDC signature network, **(E)** cDC1 vs. (pDC and cDC2) signature network, **(F)** cDC2 vs. (pDC and cDC1) signature network.

**Figure 10:**
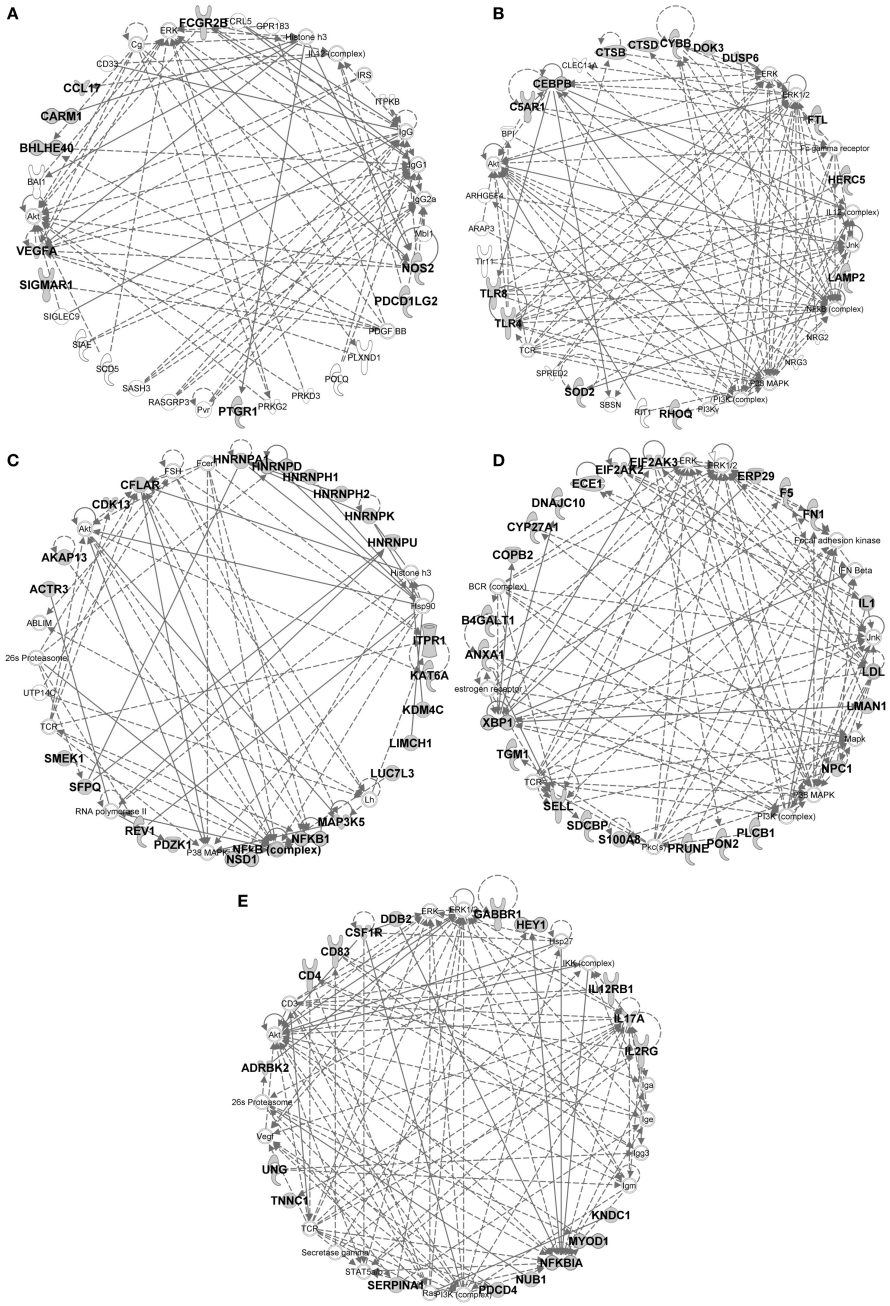
**IPA gene interaction networks of the conserved signatures in subsets of the monocytic lineage**. The conserved signature of subsets of the monocytic lineage were analyzed in Ingenuity Pathway Analysis as in Figure 9. The selected networks displayed are: **(A)** MoDC signature network, **(B)** Mo/MP vs. cDC signature network, **(C)** Mo/MP vs. MoDC signature network, **(D)** cMo vs. ncMo signature network, **(E)** ncMo vs. cMo signature network.

**Figure 11:**
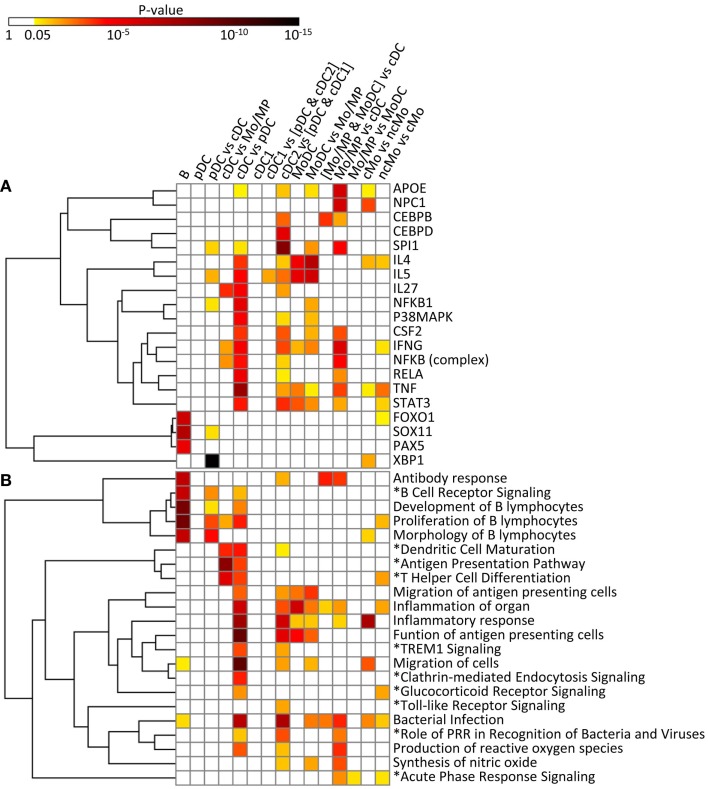
**IPA analysis of the conserved cell type gene signatures: upstream regulators (A) and biological functions and canonical pathways (B)**. A promoter sequence analysis of the conserved cell type gene signatures performed using IPA is displayed as a heatmap of the *p*-value [upstream regulators, **(A)**]. A biological function and canonical pathway analysis of the conserved cell type gene signatures performed using IPA is displayed as a heatmap of the *p*-value **(B)**. Selected upstream regulators and functions and pathways (*), in **(A,B)**, respectively, were classified using hierarchical clustering based on the average linkage metrics. Enrichments were considered significant when supported by at least three genes and by a *p*-value ≤0.05.

Taking advantage of our multi-species microarray data, we sought to identify core gene-expression signatures that should universally define at the molecular level each of the mononuclear phagocyte subset and that should hold biological relevance based on their selective and conserved expression in homologous subsets throughout mammalian evolution. Absolute signatures [“Min (test) vs. Max (ref)” method, see Materials and methods] encompassed all genes selectively expressed at higher levels in the cell subset of interest (index population) as compared to all the other cell subsets studied (comparator populations), in all species studied. An absolute signature was computed for B cells in order to validate the approach by comparison of the gene list obtained with the advanced knowledge available on the biology of this lymphocyte population. Absolute signatures were also found for pDC, cDC1, and MoDC. Relative signatures encompassed genes selectively expressed to higher levels in one or several cell subsets of interest (index population) as compared to a selection of other cell subsets (comparator populations). The choice of index and comparator populations was largely based on the branching of different cell subsets in hierarchical clustering (Figure 5), or on known sharing of specific functions between cell subsets in mouse or human. The conserved absolute and relative gene-expression signatures in mononuclear phagocyte subsets are listed in Table 1 and Data Sheet S3 in Supplementary Material. In several instances, Ingenuity Pathway Analysis (IPA) mapped a high proportion of the genes to gene interaction networks (Figure 9 for the DC lineage subsets, Figure 10 for the monocytic lineage subsets and Figure S7 in Supplementary Material), and revealed predicted upstream regulators (Figure 11A) and canonical pathways and functions (Figure 11B) that are described thereafter for B cells, DC lineage subsets, and Mo/MP categories. Although certain functions or pathways were enriched in several gene signatures, the genes responsible for the enrichments differed (Data Sheet S4 in Supplementary Material) and pointed out to different, complementary contributions of the distinct cell types to the corresponding functions or pathways.

**Table 1:** **Conserved gene signatures for mammalian mononuclear phagocytic cell subsets**.

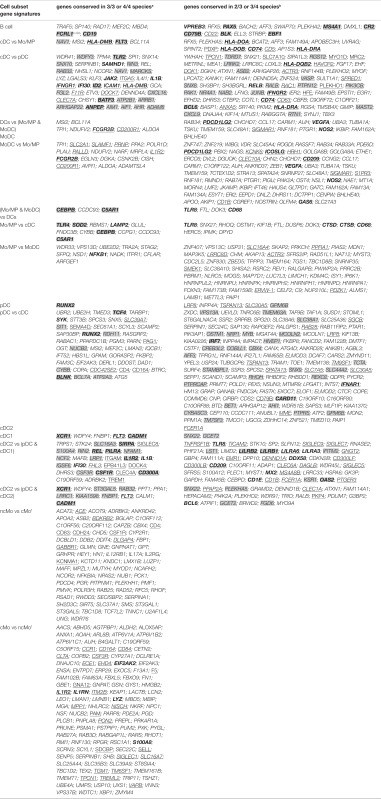

*^a^Genes conserved in 4/4 species, or in 3/3 species for cDC2, cMo, and ncMo since only three species could contribute to the analysis*.

*^b^Genes conserved in 3/4 species or 2/3 species*.

*^c^Genes in bold were previously demonstrated to play a significant role in the development or functions of the population of interest*.

*^d^Underlined genes were annotated as located in “plasma membrane” according to Ingenuity Pathway Analysis*.

*^e^Genes highlighted in gray have been previously identified as signatures genes for the corresponding mouse and human cell populations in our earlier study (15)*.

*^f^Signature genes of the relative cMo vs. ncMo and of the ncMo vs. cMo signatures were provided only for the 3/3 species selection since the gene lists for the 2/3 species selection encompassed hundreds of genes*.

The conserved B cell signature that we use as our reference subset (Table 1) includes a regulatory gene network directed to immunoglobulin production (Figure S7 in Supplementary Material), with PAX5 as an upstream regulator (*p* = 10^−5.8^) (Figure 11A). SOX11 (*p* = 10^−8^) and FOXO1 (*p* = 10^−7^) are predicted to be other upstream regulators in the conserved B signature (Figure 11A), in agreement with existing knowledge. As expected, this signature is associated to B lymphocyte ontogeny and functions [e.g., “development of B lymphocytes” *p* = 10^−10.4^, “antibody response” (*p* = 10^−7.5^), “proliferation of B lymphocytes” (*p* = 10^−10.5^), and “morphology of B lymphocytes” (*p* = 10^−7.5^) as well as to the “B cell receptor signaling” pathway (*p* = 10^−7.3^)] (Figure 11B). The B cell signature also pinpoints to genes without any known function in B cells yet, such as the cell cycle gene *RAD17* (53) or the *SP140* gene that encodes a nuclear body protein (54) (Table 1). Altogether, the results of the functional analysis of the conserved signature of B cells support the biological relevance of the conserved gene signatures generated by our approach.

In the conserved signatures corresponding to the DC lineage, the pDC signature is restricted to few genes including *RUNX2*, which encodes for a major known regulator of pDC development (56) and other genes whose role is not yet known in this subset, with three of them coding for potential cell surface markers or targeting molecules, i.e., the low density lipoprotein receptor-related protein 8 (*LRP8*), tetraspanin 13 (*TSPAN13*), and a zinc-family transporter protein member (*SLC30A5*) (Table 1). These genes, except *SLC30A5*, map to a common network (Figure 9A). No functional annotation was found significantly enriched in the pDC absolute signature due to the low number of associated genes. Interestingly, the pDC vs. cDC relative signature includes genes belonging to a regulatory network pointing to IFN −α/β production (Figure 9B) and retrieves as a major putative upstream regulator X-box binding protein 1 (*XBP1*) (*p* = 10^−15^) (Figure 11A), a transcription factor involved in mouse DC development (57). The pDC vs. cDC relative signature was also enriched for “proliferation of B lymphocytes” (*p* = 10^−4^), “morphology of B lymphocytes” (*p* = 10^−5^), and “B cell receptor signaling” pathway (*p* = 10^−2.9^), similarly to the conserved B cell signature (Figure 11B). These observations are consistent with the known usage downstream of mouse and human pDC endocytic receptors of a signaling pathway akin to that of the B cell receptor (58). This known pDC signaling pathway involves the products of *SYK*, *BLNK*, and *PIK3AP1*, three of the six genes responsible for the enrichment of the “B cell receptor signaling” pathway in the conserved pDC vs. cDC gene signature (Data Sheet S4 in Supplementary Material), as well as *CARD11* which contributes to the enrichment for the annotation “proliferation of B lymphocytes” in the pDC vs. cDC signature. This strongly suggests that this signaling pathway is conserved in pDC of all mammalian species. Beside *TCF4* which encodes for a major known regulator of both B and pDC development (52), several other genes associated to B cell biology are found in the pDC vs. cDC relative signature (Table 1), namely *CD79B*, *PTPRCAP*, *SEMA4D*, *CTCF*, *IFR1*, and *MEF2C*. This suggests that additional biological processes shared between B cells and pDC remain to be identified.

No absolute signature could be generated for cDC but interesting informations were obtained with relative signatures, i.e., the cDC vs. Mo/MP and cDC vs. pDC. The cDC vs. Mo/MP signature includes *FLT3*, a key gene in mouse DC development (59) as well as many genes of a regulatory network including *BCL11A*, *HLA-DOA*, *HLA-DRA*, *HLA-DMB*, *HLA-DOB*, *CD74*, the axone guidance neuron navigator *NAV1* and the MHC class 2 transcription regulator *RFX5* (Figure 9C). In relation to this network, *IL27* (*p* = 10^−4.4^), *IFNG* (*p* = 10^−2.6^), and NFkB (10^−2.8^) were retrieved as putative upstream regulators (Figure 11A). The cDC vs. Mo/MP signature was enriched for canonical pathways such as “antigen presentation” (*p* = 10^−9.6^), “DC maturation” (*p* = 10^−4.6^), and “T helper cell differentiation” (*p* = 10^−6.2^) (Figure 11B). The cDC vs. pDC signature includes a main regulatory network encompassing *PIK3CB*, *ICAM1*, *CLEC7A*, *HLA-DRA*, *IL1B*, and *LGALS3* (Figure 9D) and is enriched for “functions of antigen-presenting cells” (*p* = 10^−11.1^), “inflammatory response” (*p* = 10^−8.9^), “bacterial infection” (*p* = 10^−7.9^), “migration of cells” (*p* = 10^−11.4^), and “clathrin-mediated endocytosis signaling” pathway (*p* = 10^−4.6^) (Figure 11B). TNF (*p* = 10^−9^), RELA (10^−5.8^), NFKB1 (10^−6.1^), P38 MAPK (10^−5.5^), IFNG (10^−5.4^), and to a lesser extent STAT3 (10^−4.7^) and CSF2 (10^−4.3^) are predicted as putative upstream regulators in this signature (Figure 11A). In addition, this cDC vs. pDC signature includes *BATF3*, a gene highly expressed in cDC that is key in cDC1 development in mouse and human (60), as well as *ARHGAP22*, a gene involved in actin cytoskeleton regulation (61), that was initially described as a top gene of the absolute cDC signature common to human and mouse (15). Altogether, the relative gene signatures of cDC emphasize their nature of highly endocytic, motile, and expert antigen-presenting cells throughout species.

The conserved cDC1 signature encompasses genes with known contribution in the biology of this lineage, such as *XCR1*, *FLT3*, and *CADM1* (59), as well as additional genes which biological function in this subset remains enigmatic, such as the germinal center B-cell-expressed transcript 2 protein (*GCET2*), the WDFY family member 4 (*WDFY4*) whose polymorphism is associated to autoimmune diseases (62), and two intracellular trafficking proteins, a formin-binding protein (*FNBP1*) (63) and Sorting Nexin-22 (*SNX22*) (64) (Table 1). The conserved cDC1 vs. (pDC and cDC2) relative signature provides a longer list of genes belonging to an interaction network that includes *BCL6*, a transcriptional repressor that was recently found involved in the specification of cDC1 (17) as well as *XCR1* and *CALM1* (Figure 9E). IPA did not retrieve significant annotations for the cDC1 absolute or relative gene signatures. This emphasizes how little is currently known on the molecular regulation of the functions specific to cDC1s, such as cross-presentation. Future studies investigating in mouse cDC1s the functional role of the genes identified here as being part of the conserved cDC1 signatures will advance our understanding of the functions of these cells and their molecular regulation.

The absolute cDC2 conserved signature was empty. Many genes of the relative cDC2 vs. (pDC and cDC1) signature belong to a network that includes *SIRP*α (*CD172A*), a selective marker of cDC2 within the DC lineage (44), together with *CSFR1*, *TREM1*, *CLEC4A* (also known as *DCIR*), *CD1B*, and *RELB* which is known to control mouse cDC2 differentiation (65) (Figure 9F). A second network includes *ITGAM* (*CD11b*), a marker used to identify mouse cDC2, *CLEC6A*, and *IL1B* (Figure 7 in Supplementary Material). SPI1 (*p* = 10^−9.8^), CEBPD (10^−6.2^), and CEBPB (10^−3.6^) are predicted upstream regulators, as well as CSF2 (10^−3.8^), STAT3 (10^−4.4^), and IFNG (10^−4^) which were already enriched in the cDC vs. pDC signature (Figure 11A). This cDC2 relative signature also includes *IFI30*, also known as *GILT*, a lysosomal thiol reductase important in MHC class II and class I antigen processing (66, 67) (Table 1). This relative signature is enriched for “function of antigen-presenting cells” (*p* = 10^−6.2^), “inflammatory response” (*p* = 10^−6.9^), and for the pathways “TREM1 Signaling” (10^−2.5^), “Toll-like receptor canonical signaling” (10^−2.5^), and “role of pattern recognition receptors in recognition of bacteria and viruses” (*p* = 10^−3.9^) (Figure 11B). Other genes were uncovered that may be important regulators of the function of cDC2s or which product could be used to identify or target these cells, including the genes coding for plasma membrane proteins such as glycoprotein *CD300A*, the sialic binding lectin *SIGLEC8*, and the paired immunoglobin-like type 2 receptor *PILRA*. This conserved relative signature shows that within the DC lineage throughout species, cDC2 express specific networks of genes related to pathogen sensing, antigen presentation, IL-1β production, and inflammation.

In the conserved signatures corresponding to the monocytic lineage, the absolute MoDC and relative MoDC vs. Mo/MP signatures are enriched for “inflammation of organ” (*p* = 10^−6.9^ and *p* = 10^−3.3^), “function of antigen-presenting cells” (*p* = 10^−5.1^ and *p* = 10^−3.6^), and “migration of antigen-presenting cells” (*p* = 10^−3.3^ and *p* = 10^−4.3^) (Figure 11B) and encompasses *NOS2*, *CCL17*, *VEGFA*, and *FCGR2B* that map to a common major network (Figure 10A). IL-4 (*p* = 10^−5.7^) and IL5 (*p* = 10^−5.9^) are predicted regulators (Figure 11A). Among other genes of interest that had not yet been associated to MoDC are the triose phosphate isomerase *TPI1*, the NADH dehydrogenase flavoprotein *NDUFV2*, the aldolase *ALDOA*, and the *CD200R1* gene that encodes for an inhibitory cell surface receptor of MP functions (68) (Table 1). The relative MoDC vs. Mo/MP signature encompasses additional genes that participate in “migration of cells” (*p* = 10^−2.3^, with *S1PR3*, *CCL17*, and *SLC2A1*), “bacterial infection” (*p* = 10^−3.2^, with *CD1B*, *CD209*, and *FCGR2B*), and “synthesis of nitric oxide” (10^−2.5^, with *PLAU*, *IL1R2*, and *NOS2*) (Figure 11B and Data Sheet S4 in Supplementary Material). The conserved MoDC signatures indicate a dominant association of this subset to inflammation, as well as to DC functional properties when compared to Mo/MP across species.

Most of the genes in the Mo/MP vs. cDC conserved signature had been previously identified as overexpressed in murine MP, such as *TLR4*, *CEBPB*, *C5AR1*, and *SOD2* (9, 15) (Table 1 and Figure 10B). A significant proportion of the genes within this signature are related to “inflammation of organ” (*p* = 10^−2.7^), “production of reactive oxygen species” (*p* = 10^−4.4^), “synthesis of nitric oxide” (*p* = 10^−3.9^), “bacterial infection” (*p* = 10^−4.6^), “role of pattern recognition receptor in recognition of bacteria and viruses” (*p* = 10^−3.3^), and “acute phase response signaling” (*p* = 10^−2.9^) (Figure 11B). Putative upstream regulators are NPC1 (*p* = 10^−6.8^), APOE (*p* = 10^−6.8^), IFNG (*p* = 10^−6.4^), SPI1 (*p* = 10^−5.2^), and NFkB (*p* = 10^−5.1^) (Figure 11A). Additional proteins are potential transcriptional regulators of importance in Mo/MP, such as the RNA-binding protein RBMS1 and the cell cycle progression factor CCPG1. The Mo/MP vs. MoDC signature includes a gene network centered on *NFkB* and *MAP3K5* (Figure 10C). Overall, the conserved Mo/MP relative signatures support the association of Mo/MP to inflammation and oxidative stress across species.

The conserved comparative signature of cMo vs. ncMo retrieved genes belonging to a network with *IL1*, fibronectin (*FN*), *S100A8*, and *XBP1* (Figure 10D), the latter being proposed as an upstream regulator (10^−2.5^) together with *NPC1* (*p* = 10^−4^) (Figure 11A), and is strongly associated to “inflammatory response” (*p* = 10^−8.4^) (Figure 11B). The reciprocal ncMo vs. cMo signature includes a gene network with *IL17A*, *CSFR1*, *NFKBIA*, and *serpinA1* (Figure 10E), and is significantly associated to the “glucocorticoid receptor signaling pathway” (*p* = 10^−2.5^) and to some extent to the “inflammation of organ” (*p* = 10^−2.6^) (Figure 11B). These relative signatures indicate that cMo have a conserved gene program directed to strong inflammation, whereas ncMo, a poorly understood subset, might be exquisitely regulated by glucocorticoids as suggested in the literature (69, 70).

Altogether, the mononuclear phagocyte system from distantly related mammals is composed of a diversity of subsets that belong to the DC or to the Mo/MP lineage and express discriminating gene signatures involved in distinct regulatory networks and biological functions conserved through mammalian evolution. In most instances, the subset signatures also point to several unexpected genes and upstream regulators that are likely to be important in the subset biology since their selective expression pattern across subsets of mononuclear cells is conserved across species.

### Phylogenetic evidences for the existence of a gene repertoire for mononuclear phagocyte subsets in birds and bony fishes

The existence of orthologous genes of the conserved mononuclear phagocyte subset signatures in reptile/birds, fishes, and agnathans, would indicate that the genetic equipment for mononuclear phagocyte subset diversity is available in vertebrate species distant from mammals. In the case of birds and reptiles, it remains unknown whether they have pDC, cDC1, and cDC2 subsets homologous to mammals. An orthology analysis of selected genes from conserved subset signatures revealed that most genes possess a unique ortholog in birds and reptiles, with conserved synteny with human, for instance *XCR1*, *BATF3*, *RUNX2*, *TSPAN13*, and *CSF1R* (Table 2). Furthermore, these same genes also possess one or more orthologs in fish. Multiple orthologs in fish are often due to the whole genome duplication that occurred during the evolution of teleosts, and to further local duplications. Importantly, fish co-orthologs of mononuclear phagocyte subset genes are generally supported by conserved synteny. Genes duplicated in fish may have been subjected to sub-functionalization, as it is the case for many immune genes duplicated in this group of vertebrates; however, some markers have a unique counterpart in fish genomes (like *BATF3*, *RFX5*, and *CIITA*), with copy loss possibly due to detrimental effects of duplication. The case of *MHC class II* is particular: although fish *MHC class II* genes are not always considered as true orthologs of human *MHC class II* genes, their sequences show the hallmarks of *bona fide* class II antigen-presenting receptors and they likely have similar functions. For c-type lectin-like (*CLEC*) molecules, no true orthologs can be identified in fish nor in birds/reptiles, as each branch of vertebrates – even each group of mammals – shows its own set of expanded *CLEC* genes. Altogether these data show that a repertoire of conserved genes for mononuclear phagocyte subsets exists in bony fishes and reptiles, which constitutes a list of candidates for relevant markers. The presence of *BATF3* and *XCR1* are hints at possible existence of cDC1 in these species, as *BATF3* specifically controls cDC1 development in mice (71) and *XCR1* expression is strictly associated to cDC1 in several mammals (34, 36, 40, 41). In contrast, the lamprey does not have identified orthologs for many of the genes selected from the transcriptomic fingerprints of the subsets of mammalian mononuclear phagocytes (Table 2). Agnathans, including lampreys and myxines, harbor three adaptive immune cell types, each expressing a specific class of variable lymphocyte receptors, VLRC, VLRA, and VLRB, and showing transcriptomic and functional commonalities with gnathostome γδ T lymphocytes, αβ T lymphocytes, and B lymphocytes, respectively (55, 72). However, it is uncertain whether or not the activation of agnathan lymphocytes requires APCs, and if so, to which extent these cells could resemble gnathostome APCs (72). Contrary to the situation in birds and fishes, our observations do not support the existence in the lamprey of gene sets similar to those defining the transcriptomic fingerprints of the mononuclear phagocytes of mammals. Although incomplete assembly and annotation of the genome of the lamprey do not allow drawing definitive conclusions, our observations are consistent with the lack in agnathans of MHC functional homologs and of the particular proteasome machinery used by mammalian APCs for antigen processing (72). Altogether, this phylogenetic study shows that the repertoire of key genes characterizing the diversity of the mononuclear phagocytes in mammals were already present in the common ancestor of tetrapods and fishes but might be largely absent in agnathans.

**Table 2:** **Search for the existence of orthologs in reptile/birds, fishes, and agnathans for selected genes of the conserved mononuclear phagocyte subset signatures**.

	Reptiles/birds			Fishes		Agnathans (lamprey)		
Cell subset gene signatures	Gene	Orthologs	Conserved synteny	Orthologs	Conserved synteny	Orthologs	Conserved synteny
cDC vs. Mo/MP (MHC-related molecules)	*HLA-DR*	–	–	–	–	–	–
	*HLA-DM*	?	–	–	–	–	–
	*HLA-DO*	–	–	–	–	–	–
	*CD74*	+ (1)	Yes	+ (Multiple)	Yes	–	–
	*CIITA*	+ (1)	Yes	+ (1)	Yes	–	–
cDC vs. Mo/MP	*NAV1*	+ (1)	Yes	+ (Multiple)	Yes	–	–
	*RFX5*	+ (1)	Yes	+ (1)	Yes	–	–
	*BCL11A*	+ (1)	Yes	+ (Multiple)	Yes	–	–
MoMP vs. cDC	*CEBPB*	+ (1)	Yes	+ (1)	Yes	–	–
	*C5AR1*	+ (1)^a^	Yes^b^	+ (1)^c^		–	–
	*SOD2*	+ (1)	Yes	+ (1)	Yes	+ (1)	?
	*APOE*	–	–	+ (Multiple)	Yes	–	–
	*TLR4*	+ (1)	Yes	+^d^	Unclear	–	–
cDC1	*XCR1*	+ (1)	Yes	+ (Multiple)	Yes^e^	–	–
	*FLT3*	+ (1)	Yes	+ (1)	Yes	–	–
cDC vs. pDC	*BATF3*	+ (1)	Yes	+ (1)	Yes	–	–
	*ARHGAP22*	+ (1)	Yes	+ (Multiple)	Yes	+ (1)	?
	*CLEC7A*	–	–	–^f^	–	–	–
B cells	*CD79B*	+ (1)	Yes	+ (1)	Loose^g^	–	–
	*PAX5*	+ (1)	Yes	+ (1)	Yes	(+)^h^	?
	*CD19*	–	–	–	–	–	–
pDC	*RUNX2*	+ (1)	Yes	+ (1)^i^	Yes	+ (1)	?
	*TSPAN13*	+ (1)	Yes	+ (Multiple)	Yes	+ (2)	? (for both)
cDC2 vs. (cDC1 and pDC)	*IFI30/GILT*	+ (1)	Yes	+ (Multiple)	Yes	+ (1)	?
	*CSF1R*	+ (1)	Yes	+ (Multiple)	Yes	–^j^	–
	*SIRPA*	?^k^	–	–	–	–	–
	*TREM1*	–^l^	–	–	–	–	–
	*CLEC4A*	–	–	–^f^	–	–	–
	*CLEC6A*	–	–	–^f^	–	–	–
More or less cDC1-specific	*CLEC9A*	–	–	–^f^	–	–	–
	*CLNK*	+ (Turkey)	Yes	+ (1)	Yes	+ (1)^m^	?

*^a^Birds have one co-ortholog of human *C5RA1* and *C5RA2*; ^b^only in the lizard Anolis, not in available bird genomes; ^c^fish generally have one co-ortholog of human *C5RA1* and *C5RA2*; ^d^only in some species: zebrafish, catfish, and salmonids; ^e^see Ref. (36); ^f^for all CLEC, no true ortholog, each deep branch of vertebrates has its own set of expanded CLEC; ^g^the neighborhood is not conserved but zebrafish *CD79B* is close to *Arhgap27* and *Plekhm1* that are on the same human chromosome (chr17) as *CD79B* but at 20 megabases; *CD79B* genes often are not annotated in fish genomes. In zebrafish, *CD79B* is ENSDARG00000088902; ^h^a lamprey gene ortholog to *PAX5* has been identified and was selectively expressed in lamprey VLRB+ cells which resemble B lymphocytes (55); however, this gene is not identified in the current publicly available assembly of the lamprey genome; ^i^duplicated in zebrafish and cavefish; ^j^a lamprey gene is a co-ortholog to all vertebrate *CSF1R*, *PDGFR*, *KIT*, *FLT3*, etc.; ^k^in birds species, several genes are co-orthologs of all mammalian SIRPs including *SIRPA*; ^l^bird TREM-like genes are more closely related to *TREM2* rather than to *TREM1*; ^m^co-ortholog of *CLNK*, *BLNK*, and other related genes*.

## Discussion

Our computational transcriptomic meta-analysis indicates that the complex organization of the mononuclear phagocyte system shows conservation throughout distantly related mammals, a finding that appears to extend to chicken, a non-mammalian vertebrate. In the present work, by using GSEA and hierarchical clustering for unbiased pan-genomic analysis of the molecular identity of immune cell subsets across four vertebrate species, we convincingly established the existence of strong homologies between these cell types across mammals, beyond the already known existence of B cells in all species. Specifically, we could align across mammals cDC1, cDC2, pDC, MoDC, Mo/MP, and cMo vs. ncMo. In addition, we found that many of the genes that we showed to be selectively expressed in distinct mononuclear phagocyte subsets in mammals have existing orthologs in bony fishes while this appears not to be the case in lamprey. Thus, our study suggests that conserved mononuclear phagocyte subsets might exist in all gnathostomes but not in agnathans. However, this hypothesis will require to be tested experimentally, by re-examining the presence of orthologous genes in lamprey upon completion of the genome assembly and its annotation, by identifying and studying candidate mononuclear phagocyte subsets in bony fishes, and by determining whether similar cells exist in sharks, rays, and lamprey. For example, orthologous genes of the conserved mononuclear phagocyte signatures (Table 2) could be targeted by the CRISPR/Cas9 technology with a reporter gene marker in order to identify and characterize mononuclear phagocyte subsets in bony fishes (73), with for certain genes the need to test several putative orthologs in fish due to genome duplication.

The two methodologies that we used to assess subset homologies across species, i.e., hierarchical clustering and GSEA, display complementary functionalities. Hierarchical clustering on filtered, centered, reduced, and aggregated datasets has the advantage of integrating all samples together into a single analysis and of providing a global overview of the homologies between cell subsets of various species (15, 17, 18, 74, 75). However, the integration of distinct datasets requires a cross-normalization procedure which consists in a rather profound mathematical transformation of the data. The normalization procedure artificially increases the variance for genes with only small differences in their initial signal intensities between the different cell types studied. Conversely, it comparatively decreases the variance for genes with high differences in their initial signal intensities between the different cell types studied. To limit the biases that this normalization introduces, it is thus necessary to select only the orthologous genes that vary strongly in their expression across the cell types examined within each species. Another corollary is that this analysis can only be applied to genes that have known orthologs in all species. If one ortholog is missing in only one species, the gene must be removed from the analysis. Hence, this method should be used with caution, only under conditions where dataset normalization does not yield too strong biases in gene-expression profiles. It is also not appropriate when the structures of the different datasets are too different (i.e., the number and potential identities of cell types vary too much across datasets), because the dynamic ranges of gene expression between datasets are not expected to be the same and should therefore not be forced to similarity. Even under conditions where the experimental design is favorable to the use of hierarchical clustering, GSEA ensures of the robustness of interpretation. GSEA has been used by us and others to perform cross-species comparisons (5, 19, 29, 42, 76–78). GSEA notably displays advantages and drawbacks distinct from those of hierarchical clustering. First, it is easier to perform GSEA since dedicated ready-to-use stand-alone programs are available which do not require bio-informatics expertise. Second, GSEA is more sensitive, notably to detect overlaps of common functions/gene networks between cell populations or cellular contaminations, as exemplified with sheep *pDC enriched in human and mouse B cell fingerprints. This higher sensitivity is linked to (i) the fact that GSEA can detect coordinate regulation of gene modules (geneset-based approach) and thus does not rely on the strong regulation of few single genes (single gene-based approach), (ii) the fact that GSEA, when applied to multiple species, takes into account all genes that have orthologous counterparts in the considered species and is not restricted only to highly variable genes. Third, GSEA can perform cross-platform comparison without any cross-normalization thus without any supplementary artificial manipulation of the expression data. Finally, it can be performed on multiple datasets, even if their structures are different. However, GSEA presents the limitation of performing pairwise comparisons whose results can be integrated and visualized with our Bubble GUM software, but it nevertheless does not provide a global trans-species overview of subset homology. Overall, in order to increase confidence in the interpretation of the results, it is important to combine both approaches and verify that they both lead to consistent conclusions.

Our subset assignment methodology demonstrates similarity or proximity between subsets across species but not strict identity. Besides possible intrinsic transcriptomic differences between species, one of the reasons that explain this limitation is the process of subset identification itself, which makes use of different surface markers. Whenever possible, similar marker combinations were used such as CADM1 and CD172 that are known to be conserved markers across human, mouse, and sheep cDC subsets (42). However, mAb anti-CD11c did not exist for the initial gating in pig and the mAbs in the exclusion pool were not the same in pig and sheep. Moreover, existing marker combinations are not always specific and can lead to cross-contamination between different cell subsets. Indeed, the GSEA of the sheep *cDC2 revealed that they may have been contaminated by pDC, despite our attempt to avoid this problem through exclusion of CD45RB-expressing cells. It remains possible that pDC expressing minimal levels of CD45RB were still present in the sorted *cDC2 population, and not in the sheep cDC1 subset. However, since sheep *cDC2 were found in the correct cDC branch of the hierarchical clustering, their contamination by pDC is likely to have been limited. Similarly, it is likely that the sorted sheep *pDC include residual B cells, explaining the enrichment for the human B cell fingerprint at a level above expectation: indeed after exclusion of B cells with a pan-B cell marker, sheep *pDC were selected with a mAb directed to CD45RB, which may react with residual B cells that have escaped the pan-B cell exclusion. Yet, sheep *pDC still cluster with other species pDC, separately from B cells. In the case of pig, pDC were selected using markers not expressed by B cells and they displayed an enrichment for B cell fingerprints at a level encountered in GSEA analyses of mouse pDC (Figure 4 in Supplementary Material). Finally, our approach was able to demonstrate that *a priori* assignment of subset identity based on the expression of a few membrane markers could be wrong, like in the case of the pig *cDC2. Moreover, our approach had the power to properly re-assign cell subset identity, demonstrating that pig *cDC2 were actually homologous to mouse and human ncMo. Another laboratory analyzed the transcriptome of similar pig cells sorted as CD14^low^ CD163^high^ cells, but they could not assign them to classical nor to non-classical human Mo, due to differences in bio-informatics approaches in this study (79) and in ours.

Our study will help improving in the near future the toolbox available in each species for rigorous and consistent phenotypic identification of cell subsets, thanks to our identification of novel, conserved, and specific, combinations of surface markers for each cell subset, which should allow generating more appropriate staining reagents. For instance, fluorescently labeled recombinant XCL1 could theoretically be used in any species to rigorously identify and sort cDC1 (38, 41). In addition, cell surface proteins encoded by genes shown here to be selectively expressed in a conserved manner in specific subsets of mononuclear phagocytes represent new candidate markers to refine and homogenize phenotypic identification of these cells across species, such as LRP8, TSPAN13, NRP1, and SLC30A5 for pDC, FCGR2B, and CD200R1 for MoDC, SIGLEC8 and IGSF6 for cDC2, and CSF1R, TLR4, and C5AR1 for Mo/MP (Table 3). However, these potential new markers for subset identification need to be validated at the protein level.

**Table 3:** **Proposition of marker combination for oligo-phenotyping of mononuclear phagocytic cell subsets across species**.

Exclusion	Anti-CD3, anti-NK cells, and anti-B cells, if available^a^						
Targeted cell population	pDC	cDC1	cDC2	MoDC	cMo	ncMo	MP
Combination of known markers^b^	FLT3^+^	FLT3^hi^	FLT3^+^	FLT3^−^	FLT3^−^	FLT3^−^	FLT3^−^
	SIRPα^lo^	SIRPα^lo^	SIRPα^+^	SIRPα^+^	SIRPα^+^	SIRPα^+^	SIRPα^+^
	MHC-II^lo^	MHC-II^+^	MHC-II^+^	MHC-II^+^			
		CD11c^+^	CD11c^+^	CD11c^+^			
		CADM1^hi^	CADM1^lo^				
New additional candidates^c^	LRP8^+^	XCR1^+^	SIGLEC8^+^	FCGR2B^hi^	CSF1R^int^	CSF1R^hi^	CSF1R^+^
	TSPAN13^hi^		IGSF6^+^	CD200R1^+^	CCR1^+^	CD83^+^	TLR4^hi^
	SLC30A5^+^				C19ORF59^+^		C5AR1^+^
	NRP1^+^						

*^a^ Exclusion with anti-CD3, anti-NK cells, and anti-B cell markers is desirable when appropriate tools are available*.

*^b^ A combination of known markers including FLT3, MHC-II, CD11c, SIRPα, and CADM1 allows a first step of identification of subset candidates but is at risk of contamination by sister cell types, or may be incomplete due to non-availability of one of the marker. FLT3 labeling may be performed by using recombinant His-tag FLT3L generated for the relevant species as recently proposed in a review (21)*.

*^c^ New additional candidate markers for refinement of subset identification are derived from the identification of genes encoding cell surface molecules from the conserved cell subset gene signatures*.

The subset-specific signatures that are conserved throughout distant mammals included variable number of genes that were sometimes far lower than the numbers of genes in the human/mouse common signatures. There are several explanations to this finding. There is a contribution of the very high stringency of the “Min (test) vs. Max (ref)” ≥1x method that we used to establish the signatures, since any gene which was not consistently found overexpressed in all the replicates of all the species was excluded. As an example, the gene *DNAJC7*, identified as specific of pDC in our previous work (15) was removed from the human pDC signature because its “Min (test) vs. Max (ref)” ratio was equal to 0.933, due to a single lower human pDC replicate compared to a single replicate found with a higher signal in human MoDC. There is also a contribution of incomplete mapping of the genome of some of the species studied, leading to an underestimation of the number of orthologous genes that could be queried across all species. For example, *POU2F2*, more highly expressed in human and murine B cells as compared to many other immune cells, has not been mapped yet to the pig genome while it has been mapped to the genome of more distant species such as the spotted gar with a 1-to-1 orthology relationship. Another prominent cause is linked to technical limitations of the microarray approach, such as lack of ProbeSets against certain genes in certain species. This is notably the case for the gene *CLEC9A*, known to be specific of cDC1 but for which no ProbeSet exists in the human Affymetrix HG U133 plus2 gene chip. Sometimes, low signal-to-noise ratio for certain ProbeSets can also be responsible for the loss of putative interesting signature genes, such as *ZNF521* (*Zfp521* in mouse) found to be highly specific of pDC in mouse and human while the pig and sheep orthologous ProbeSet remains at the background level whatever the cell type considered. Recent technological advances now allow performing high throughput RNA sequencing at single cell levels with high sensitivity and processivity, which could solve most of the above issues; indeed, all expressed genes should be detected without any bias and analysis at the single cell level should alleviate any issue of cross-contamination between cell types. Therefore, the generation of gene-expression data for many individual cells of the same type should increase statistical power to define genes co-expressed at the single cell level and defining cell type-specific transcriptomic modules (22). Single cell gene-expression profiling recently allowed the unbiased and *de novo* identification of the different cell types of spleen (80) and central nervous system (81, 82) via the description of their molecular identity, starting from the bulk population of all the cells that could be extracted from the organ, without any prior enrichment procedure, based on the use of potentially confounding phenotypic marker combinations. However, this strategy is still extremely difficult to apply to species which genome has not yet been completely assembled, as well as to very rare cell types recovered upon prior phenotype-based enrichment. Moreover, to obtain information of sufficient completeness on functionally important genes for which few mRNA are expressed per cell, it is necessary to sequence at a sufficient depth of about one million reads per cell, which today still represents a very high cost when multiplied by the number of individual cells and conditions. Finally, the interpretation of the RNA-seq data on single cells is still largely based on the transcriptomic/molecular identity of cell types that are deduced from microarray analysis of purified cell pools. Hence, our work constitutes a major advancement in the field and is a necessary step before an eventual, later, refinement of the definition of cell subsets and their associated molecular signatures using single cell RNA-seq. The canonical gene-expression signatures that we generated can be used to distinguish and identify cell subsets in other vertebrate species. The cDC1 signature and the cDC2 vs. cDC1 signatures could be evaluated in chicken cDC sorted as single cells to determine whether this population includes only cDC1, as suggested by the trans-vertebrate hierarchical clustering, or a mixture of cDC1 and cDC2.

The conservation of gene signatures and interacting gene networks in homologous cell subsets throughout evolution is likely to bear strong biological meaning. Indeed, many genes of the conserved signatures were already known for their functions in these cells, validating the biological relevance of our signatures. In several instances, the same functional annotations were enriched in distinct subset signatures, but the genes responsible for the enrichments differed. For example, the genes responsible for the enrichment of the pathway “role of pattern recognition receptor in recognition of bacteria and viruses” were *TLR4*, *TLR8*, and *C5AR1* for the Mo/MP vs. cDC signature, *TLR2*, *CLEC7A*, *IL1B*, and *PIK3CB* for the cDC vs. pDC signature, and *TLR8*, *CLEC6A*, *DDX58*, *OAS2*, and *IL1B* for the cDC2 vs. (pDC and cDC1) signature. This analysis shows that cDC and MP express different sets of pattern recognition receptors for detection of viruses and bacteria, and that, within DC, cDC2 are also equipped differently from cDC1 and pDC for sensing of viruses and bacteria. These observations extended to other mammalian species the previous reports that human and mouse cDC2 are preferentially equipped with PRR targeting bacteria or involved in cytosolic sensing of viral infection (83, 84), and that TLR4 is very weakly expressed on pDC and cDC as compared to Mo/MP (83, 85). Similarly, different subset signatures were all enriched for “inflammatory response,” “inflammation of organs,” and “bacterial infection” but due to different genes. Altogether, this analysis indicates that different mononuclear phagocyte subsets express distinct and specific gene-expression modules which can sometimes contribute in a complementary way to the same general biological process in a conserved manner throughout evolution. Within the conserved gene-expression programs in mononuclear phagocyte subsets, we identified novel candidate genes and putative upstream regulators which likely contribute to the control of the ontogeny or functions of the corresponding cell type. For instance, the *FNBP1* and *SNX22* encoded proteins may be involved in the specific intracellular trafficking properties promoting antigen cross-presentation by cDC1, *ARHGAP22* and *NAV1* could modulate the organization of the cytoskeleton of cDC to control their mobility or antigen presentation functions, and the transcription regulators *BCL11A* and *MSL2A* may control specific gene networks in cDC. *BLC11A* is known to be key in murine pDC development (50) but it may have a specific role in cDC homeostasis, as inferred from a previous study (86). Our study thus opens the way for deciphering the sets of genes encoding functional cellular modules and their specifying transcription factors in subsets of mononuclear cells, in order to further improve and connect together the molecular and functional definitions of these cell types across species (22, 23).

## Conclusion

Our meta-analysis that combines cell sorting and comparative transcriptomic analysis was implemented as a methodology pipeline that could be used by biologists with minimal training in bio-informatics for subsequent extension to other species and to other complex cellular systems. Our study should lead to the identification of homologous mononuclear phagocyte subsets in species other than sheep and pigs, and which are of importance for biomedical investigations, such as bats, rabbits, ferrets, guinea pigs, possibly zebrafishes, and in species of veterinary importance including pets and animals of the food economy. The characterization of mononuclear phagocyte subsets in these species will allow manipulating their immune responses against diseases for the sustainability of our environment.

## Author Contributions

ISC and MD directed research and wrote the paper with input from TPVM. TPVM carried out bio-informatics analyses with input from MD. JEY performed most of the cell purification experiments and analyzed data, with input from CU (microarray hybridization, blood processing), SR (pDC isolation), MB (cell sorting), MM (microarray hybridization and analysis), HM (chicken array data), PQ (chicken array data), NB (pig cell phenotyping). PB performed phylogenetic analyses. GF and HS provided key cell types (MoDC) and reagents (unique mAb, non-commercially available). LJ performed array annotations.

## Conflict of Interest Statement

The authors declare that the research was conducted in the absence of any commercial or financial relationships that could be construed as a potential conflict of interest.

## Supplementary Material

The Supplementary Material for this article can be found online at http://journal.frontiersin.org/article/10.3389/fimmu.2015.00299

## Footnotes

^1^http://crb-gadie.inra.fr/

^2^http://www.ciml.univ-mrs.fr/applications/BubbleGUM/index.html
