# Erratum: Defining Mononuclear Phagocyte Subset Homology across Several Distant Warm-Blooded Vertebrates through Comparative Transcriptomics

**DOI:** 10.3389/fimmu.2016.00143

**Published:** 2016-04-28

**Authors:** Frontiers Production Office

**Affiliations:** ^1^Frontiers Production Office, Frontiers, Switzerland

**Keywords:** comparative biology, immunology, dendritic cells, monocytes, macrophages, genomic and bio-informatic methods

**Reason for Erratum:**

Due to a typesetting error, a misalignment in Table 1 lead to the publication of incorrect information. The publisher apologizes for this error and the correct version of Table 1 appears below.

**Table 1:** **Conserved gene signatures for mammalian mononuclear phagocytic cell subsets**.

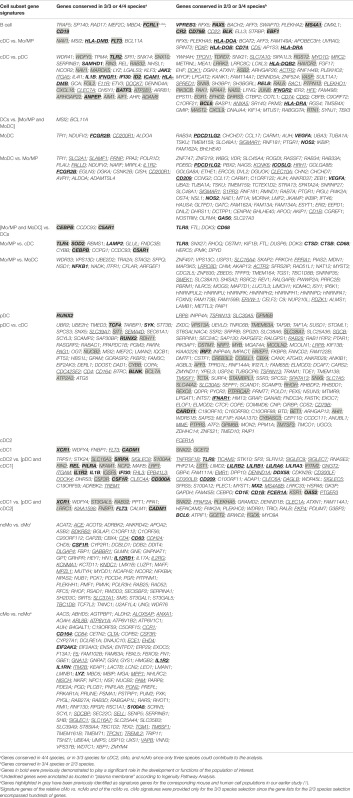

This error does not change the scientific conclusions of the article in any way.
