# Supplementary material for: Defining Mononuclear Phagocyte Subset Homology Across Several Distant Warm-Blooded Vertebrates Through Comparative Transcriptomics
Source: Front Immunol. 2015 Jun 19;6:299. doi: 10.3389/fimmu.2015.00299 (PMC4473062; doi:10.3389/fimmu.2015.00299)
Supplement: Supplementary file 1 [file table_1.docx]

**Suuplementary table 1. List of primers used to detect expression of myeloid genes in artiodactyl subsets**

| Primer name  F forward, R reverse | Sequence |
| --- | --- |
| **Pig** | |
| TCF4-F | TTTGCCATCTTCCGTTTATGC |
| TCF4-R | CCGGGCGAGTCCCTATTG |
| FLT3-F | TGTTCACGCTGAATATAAGAAGGAA |
| FLT3-R | GGAGCAGGAAGCCTGACTTG |
| BATF3-F | TCAAGCATCACTGTGCATCCA |
| BATF3-R | GGAGGACATGATTCTGCACAAC |
| XCR1-F | CGATGCCGTCTTCCACAAG |
| XCR1-R | GGAACCACTGGCGTTCTGA |
| CSF1R-F | TGAACGACTCCAACTACATTGTCA |
| CSF1R-R | TGTAGACGCAGTCGAAGATGCT |
| **Sheep** | |
| TCF4-F | TGGTCTGGCCTCAGGGTATG |
| TCF4-R | GGCCCCAACCATGAGTGA |
| FLT3-F | TGTTCACGCTGAATATAAGAAGGAA |
| FLT3-R | GGAGCAGGAAGCCTGACTTG |
| BATF3-F | CGAACATGAGCCCTGAGGAT |
| BATF3-R | CTGAGCAGCAACTCGGTTTTT |
| XCR1-F | TGCCATCTTCCACAAGGTGTT |
| XCR1-R | ACGGAGGCGAGGAACCA |
| ZBTB46-F | CACAGCCCAGGGCTTCAA |
| ZBTB46-R | ATGACGTTCCTGCTGGTGAGA |
| CD14-F | TGAACATTGCCCAAGCACAC |
| CD14-R | GCCGAGACTGGGATTGTCAG |
| GADPH-F | CCTGGAGAAACCTGCCAAGT |
| GAPDH-R | GCCAAATTCATTGTCGTACCA |
