# Supplementary material for: Defining Mononuclear Phagocyte Subset Homology Across Several Distant Warm-Blooded Vertebrates Through Comparative Transcriptomics
Source: Front Immunol. 2015 Jun 19;6:299. doi: 10.3389/fimmu.2015.00299 (PMC4473062; doi:10.3389/fimmu.2015.00299)
Supplement: Supplementary file 6 [file image_1.pdf]

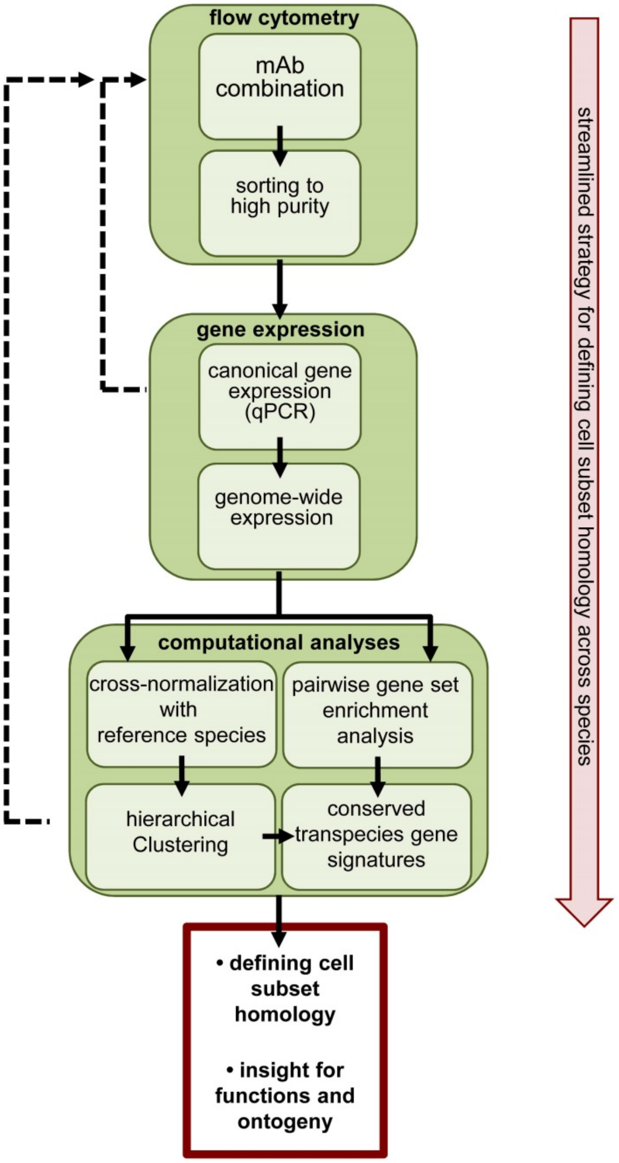

Supplementary Figure 1. Strategy for identification of homologous subsets across species. The strategy includes three main successive steps, namely flow cytometry, gene expression data collection and computing analyses. Flow cytometry: i) antibody panels are designed for putative identification of cell subsets in each species according to conserved markers between mice and men and to reagents available in each species and ii) candidate cell subsets are sorted to high purity by using advanced multiparameter flow cytometry. Gene expression data: i) initial quality controls are performed by assessing canonical gene expression using qPCR in order to a priori identify cell subset candidates and refine the antibody panels used for cell subset sorting and ii) genome-wide transcriptomic profiles are generated for the sorted cell subsets. Computing: a posteriori computational transcriptomic analyses are performed to establish the homology of the sorted candidate cell subsets with human and mouse reference cell subsets by using GSEA and hierarchical clustering. In some cases, i) the a priori assignment of candidate subsets can be wrong and the a posteriori analysis can lead to switch assignment of cell type homology, ii) the computing analysis may lead refinement of the antibody panels used for cell subset sorting, or iii) homology may not be clearly established. Conserved cell subset transcriptomic signatures consist in the genes that are differentially expressed in well-described cell subsets from a referent species and in the homologous subsets of other species. They encompass newly identified candidate genes with potentially important and conserved roles in the ontogeny and functions of cell subsets.
