# Supplementary material for: Defining Mononuclear Phagocyte Subset Homology Across Several Distant Warm-Blooded Vertebrates Through Comparative Transcriptomics
Source: Front Immunol. 2015 Jun 19;6:299. doi: 10.3389/fimmu.2015.00299 (PMC4473062; doi:10.3389/fimmu.2015.00299)
Supplement: Supplementary file 7 [file image_2.pdf]

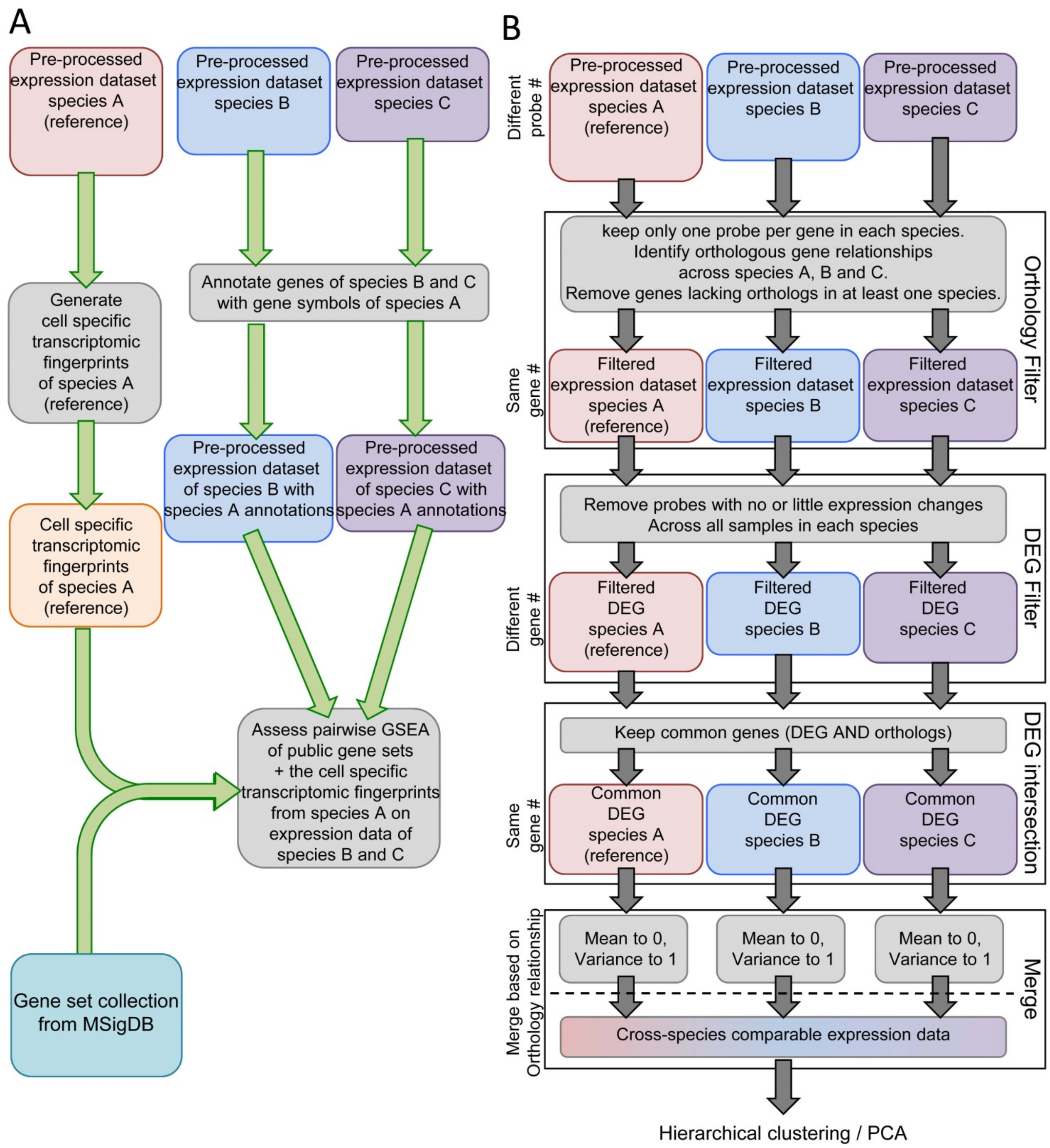

Supplementary Figure 2. Computational analyses to assess inter-species transcriptomic similarity between cell types. The pre-processed microarray expression data coming from different species (A, B and C) thus from different platforms (illustrated by boxes of different sizes because of the different probe numbers) and corresponding to different cell types are compared through two independent methods. A) Cross-species transcriptome comparison by pairwise GeneSet Enrichment Analyses. This method tests the conservation of the transcriptomic fingerprints of each cell type from the referent species (species A, i.e. the species for which the cell types are the most accurately described and generally for which gene orthologous relationships can be retrieved from) within the expression data of the samples from species B and C. B) Cross-normalization of species-specific expression datasets. This method offers the possibility to perform classification algorithm such as hierarchical clustering, Self-Organizing Map (SOM) and principal component analysis. See material and methods.
