# Supplementary material for: Defining Mononuclear Phagocyte Subset Homology Across Several Distant Warm-Blooded Vertebrates Through Comparative Transcriptomics
Source: Front Immunol. 2015 Jun 19;6:299. doi: 10.3389/fimmu.2015.00299 (PMC4473062; doi:10.3389/fimmu.2015.00299)
Supplement: Supplementary file 8 [file image_3.pdf]

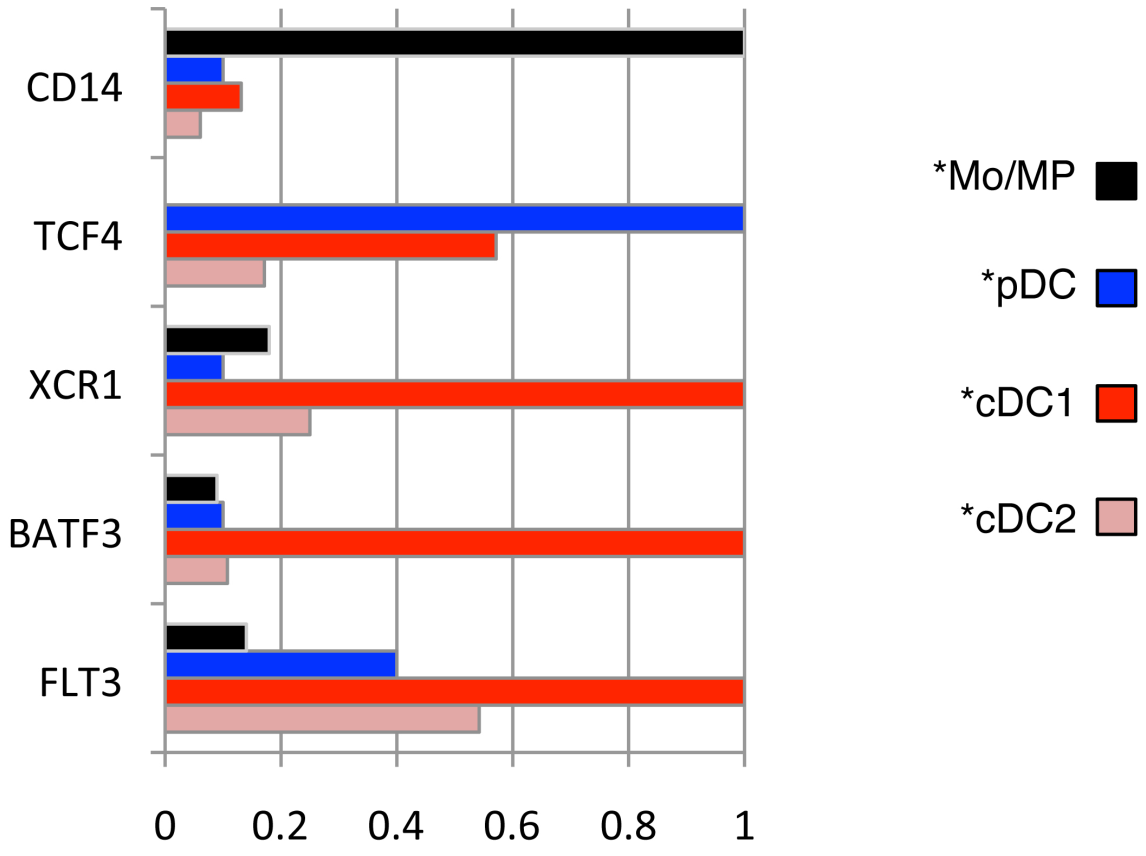

Supplementary Figure 3. Analysis of expression of control genes in initial sorting of cDC candidates without CD45RB$^+$ cell exclusion. Sheep *cDC1 and *cDC2 candidates were sorted from blood as described in Fig. 2 except that the CD45RB$^+$ cell exclusion was omitted. The expression of control genes shows a strong expression of TCF4 especially in the cDC1 candidate, indicating contamination of this population by pDC.
