# Supplementary material for: Defining Mononuclear Phagocyte Subset Homology Across Several Distant Warm-Blooded Vertebrates Through Comparative Transcriptomics
Source: Front Immunol. 2015 Jun 19;6:299. doi: 10.3389/fimmu.2015.00299 (PMC4473062; doi:10.3389/fimmu.2015.00299)
Supplement: Supplementary file 9 [file image_4.pdf]

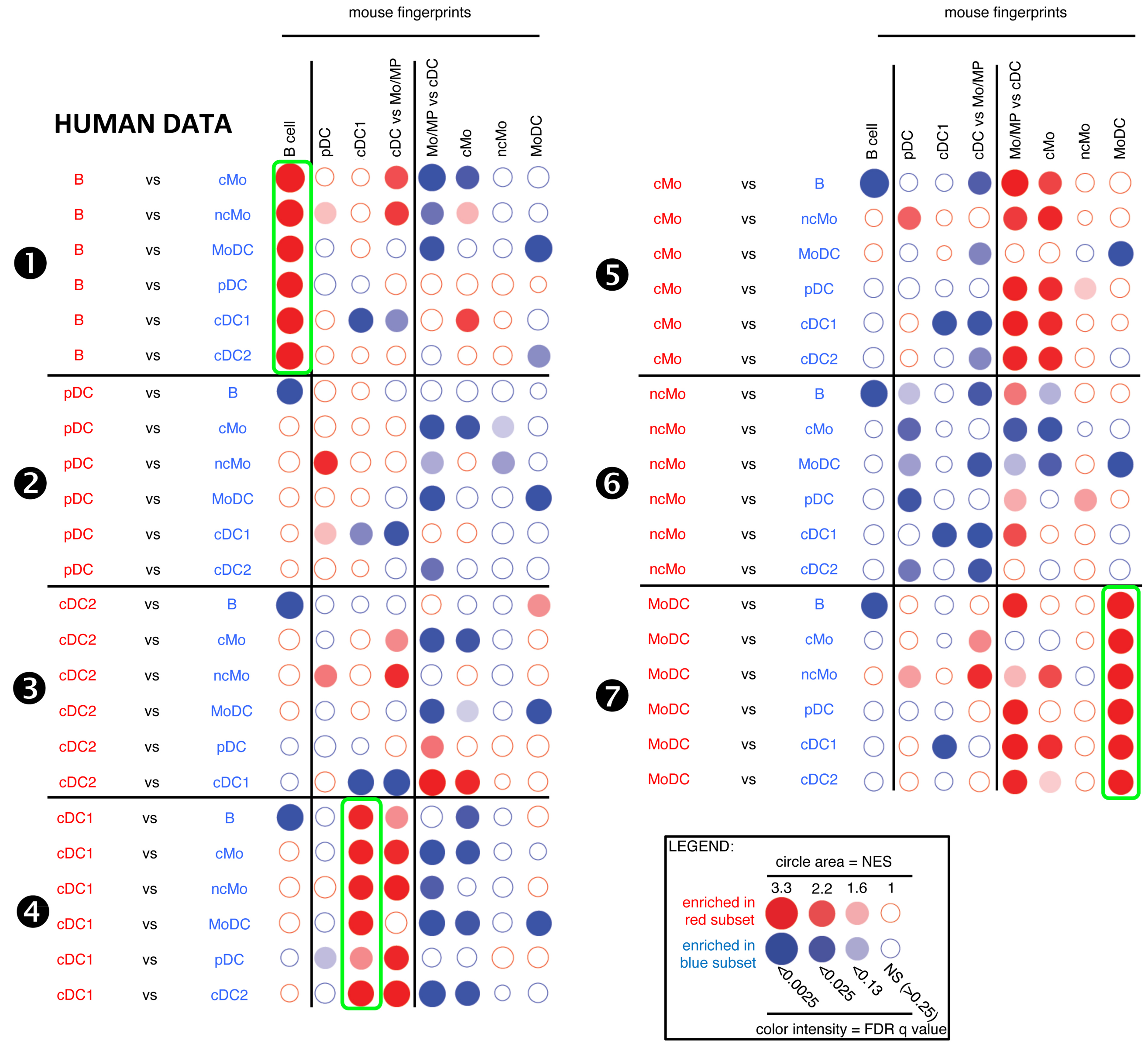


**Supplementary Figure 4. Analysis of the conservation of murine cell-subset transcriptomic fingerprints into their human equivalent cell subsets using GSEA.** GSEA was performed using sets of genes corresponding to the transcriptional fingerprints of murine B cells, pDC, cDC1, cMO (Ly6C$^{+}$ MHC class II$^{-}$) and ncMo (Ly6C$^{-}$ MHC class II$^{-}$), MoDC as compared to many other leukocytes, or corresponding to genes found more highly expressed in cDC as compared to Mo/MP or inversely more highly expressed in Mo/MP as compared to cDC. Pair-wise comparisons of human expression data were performed to assess enrichment of the murine GeneSets. Results were analyzed and represented exactly as in Figure 3.
