# Supplementary material for: Defining Mononuclear Phagocyte Subset Homology Across Several Distant Warm-Blooded Vertebrates Through Comparative Transcriptomics
Source: Front Immunol. 2015 Jun 19;6:299. doi: 10.3389/fimmu.2015.00299 (PMC4473062; doi:10.3389/fimmu.2015.00299)
Supplement: Supplementary file 10 [file image_5.pdf]

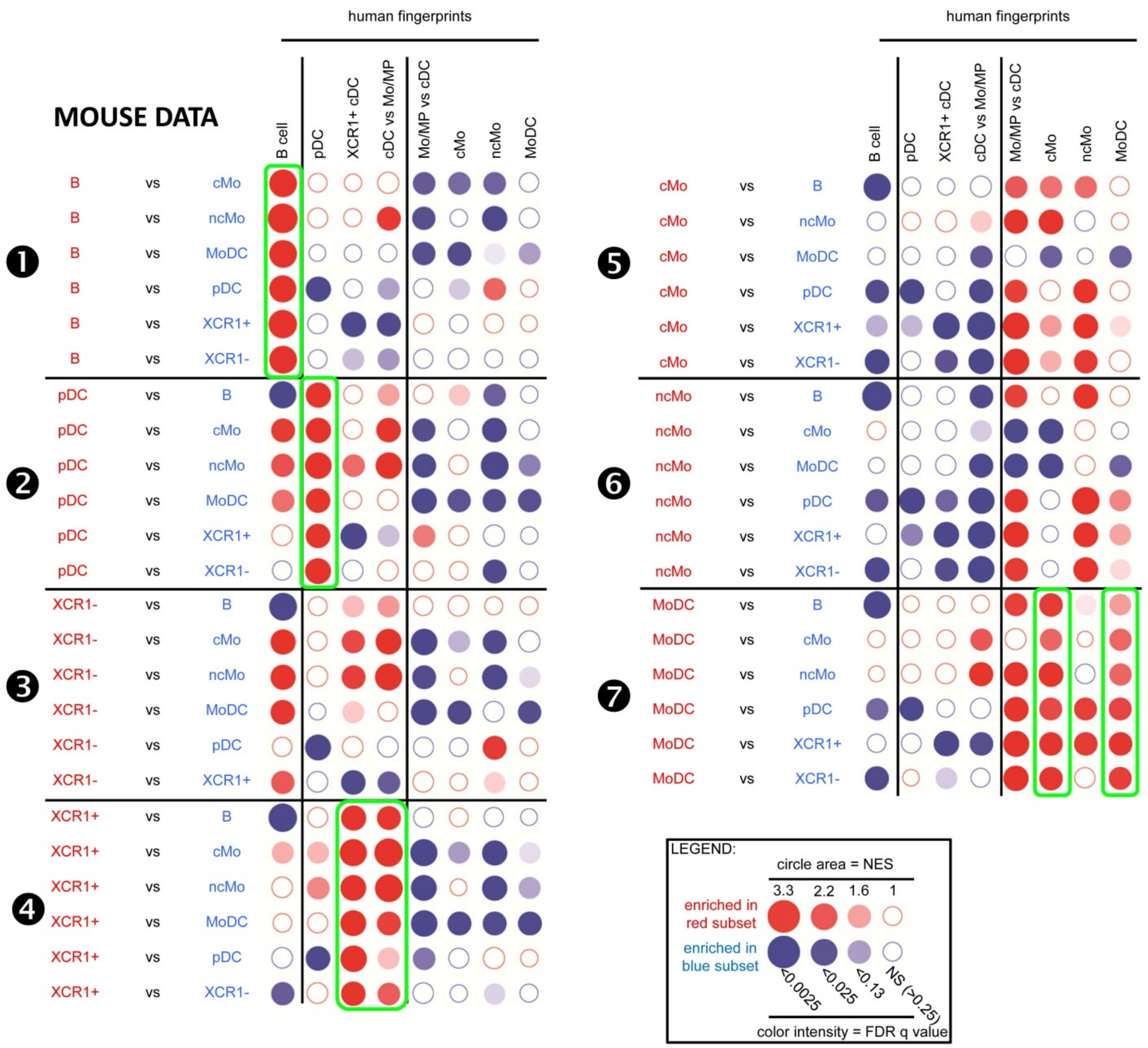

Supplementary Figure 5. Analysis of the conservation of human cell-subset transcriptomic fingerprints into their murine equivalent cell subsets using GSEA. GSEA was performed using sets of genes corresponding to the transcriptional fingerprints of human B cells, pDC, XCR1^{+} cDC, cMO (CD14^{+}), ncMo (CD16^{+}) and MoDC as compared to many other leukocytes, or corresponding to genes found more highly expressed in cDC as compared to Mo/MP or inversely more highly expressed in Mo/MP as compared to cDC. Pair-wise comparisons of murine expression data were performed to assess enrichment of the human GeneSets. Results were analyzed and represented exactly as in Figure 3.
