# Supplementary material for: Defining Mononuclear Phagocyte Subset Homology Across Several Distant Warm-Blooded Vertebrates Through Comparative Transcriptomics
Source: Front Immunol. 2015 Jun 19;6:299. doi: 10.3389/fimmu.2015.00299 (PMC4473062; doi:10.3389/fimmu.2015.00299)
Supplement: Supplementary file 11 [file image_6.pdf]

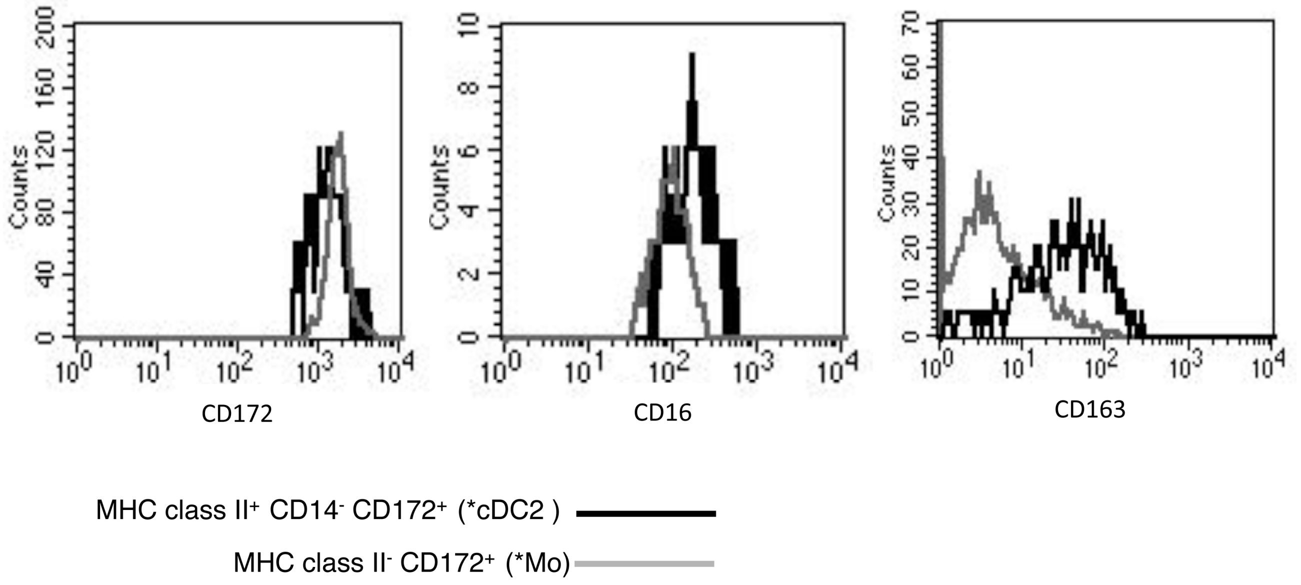

Supplementary Figure 6. Analysis of the surface expression of CD172, CD16 and CD163 on pig MHC class II$^+$ CD14$^-$CD172$^+$ (*cDC2) and MHC class II$^-$ CD172$^+$ cells (*Mo). Low density PBMC selected as MHC class II$^+$ CD14$^-$ CD172$^+$ (black line) and MHC class II$^-$ CD172$^+$ cells (grey line) were analyzed for CD172, CD16 and CD163 expression.
