# Supplementary material for: Defining Mononuclear Phagocyte Subset Homology Across Several Distant Warm-Blooded Vertebrates Through Comparative Transcriptomics
Source: Front Immunol. 2015 Jun 19;6:299. doi: 10.3389/fimmu.2015.00299 (PMC4473062; doi:10.3389/fimmu.2015.00299)
Supplement: Supplementary file 12 [file image_7.pdf]

B cell-specific

cDC2 vs [pDC & cDC1] 2nd network

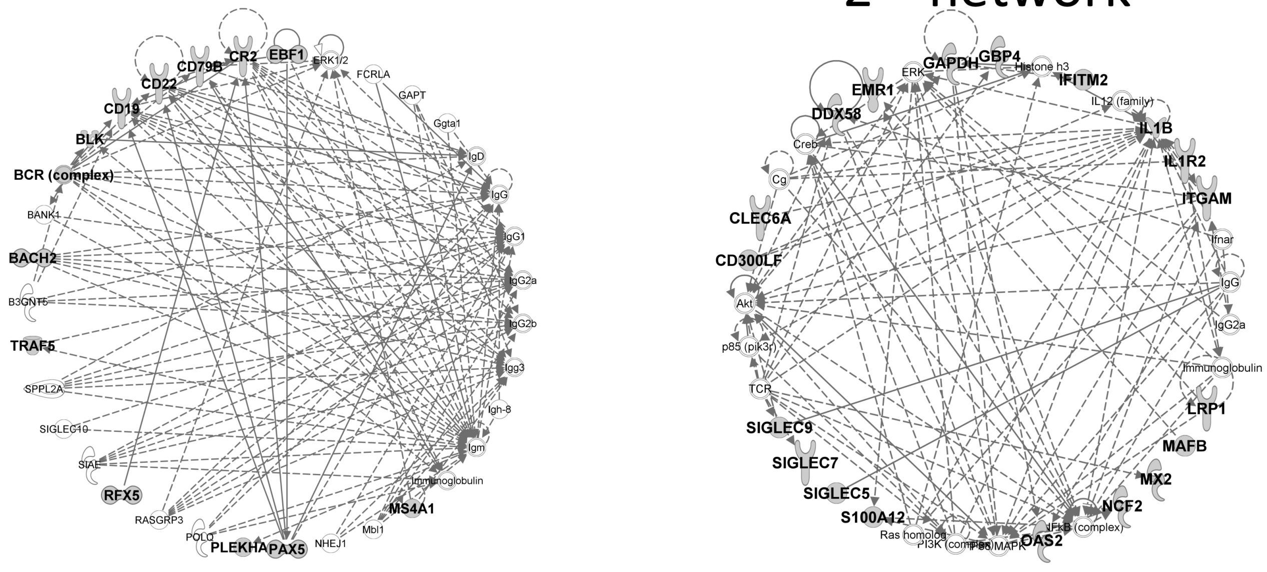

Supplementary Figure 7. IPA gene interaction networks of the conserved B cell-specific and cDC2 vs [pDC & cDC1] signatures. The cell type signatures were analyzed in Ingenuity Pathway Analysis which generates networks based on the connectivity of the genes in each signature (in boldface) but also on their connectivity with genes not belonging to the signature (in plain characters). The identified networks are displayed as graphs showing the molecular relationships between genes/gene products. Genes are represented as nodes, and the biological relationship between two nodes is represented as an edge (line). The edges can represent direct (continuous) or indirect (dashed) relationships between nodes.
